# Ionothermal synthesis of open-framework metal phosphates with a Kagomé lattice network exhibiting canted anti-ferromagnetism†
†Electronic supplementary information (ESI) available: Cif files, atomic parameters, X-ray diffraction patterns, IR spectra, TG curves, and thermal ellipsoid plot and atomic label schemes of compound **1–4**. See DOI: 10.1039/c4tc00290c



**DOI:** 10.1039/c4tc00290c

**Published:** 2014-08-04

**Authors:** Guangmei Wang, Martin Valldor, Bert Mallick, Anja-Verena Mudring

**Affiliations:** a Inorganic Chemistry III – Materials Engineering and Characterization , Ruhr-Universität Bochum , D-44780 Bochum , Germany; b Department of Materials Science and Engineering , Iowa State University, and Critital Materials Institute , Ames Laboratory , Ames , IA , USA; c Physics of Correlated Matter , Max Plank Institute for Chemical Physics of Solids , D-01187 Dresden , Germany

## Abstract

Four open-framework transition-metal phosphates; (NH_4_)_2_Co_3_(HPO_4_)_2_F_4_ (**1**), (NH_4_)Co_3_(HPO_4_)_2_(H_2_PO_4_)F_2_ (**2**), KCo_3_(HPO_4_)_2_(H_2_PO_4_)F_2_ (**3**), and KFe_3_(HPO_4_)_2_(H_2_PO_4_)F_2_ (**4**); are prepared by ionothermal synthesis using pyridinium hexafluorophosphate as the ionic liquid. Single-crystal X-ray diffraction analyses reveal that the four compounds contain cobalt/iron–oxygen/fluoride layers with Kagomé topology composed of interlinked face-sharing MO_3_F_3_/MO_4_F_2_ octahedra. PO_3_OH pseudo-tetrahedral groups augment the [M_3_O_6_F_4_] (**1**)/[M_3_O_8_F_2_] layers on both sides to give M_3_(HPO_4_)_2_F_4_ (**1**) and M_3_(HPO_4_)_2_F_2_ (**2–4**) layers. These layers are stacked along the *a* axis in a sequence AA…, resulting in the formation of a layer structure for (NH_4_)_2_Co_3_(HPO_4_)_2_F_4_(**1**). In NH_4_Co_3_(HPO_4_)_2_(H_2_PO_4_)F_2_ and KM_3_(HPO_4_)_2_(H_2_PO_4_)F_2_, the M_3_(HPO_4_)_2_F_2_ layers are stacked along the *a* axis in a sequence AA_*i*_… and are connected by [PO_3_(OH)] tetrahedra, giving rise to a 3-D open framework structure with 10-ring channels along the [001] direction. The negative charges of the inorganic framework are balanced by K^+^/NH_4_^+^ ions located within the channels. The magnetic transition metal cations themselves form layers with stair-case Kagomé topology. Magnetic susceptibility and magnetization measurements reveal that all four compounds exhibit a canted anti-ferromagnetic ground state (*T*_c_ = 10 or 13 K for Co and *T*_c_ = 27 K for Fe) with different canting angles. The full orbital moment is observed for both Co^2+^ and Fe^2+^.

## Abstract 2

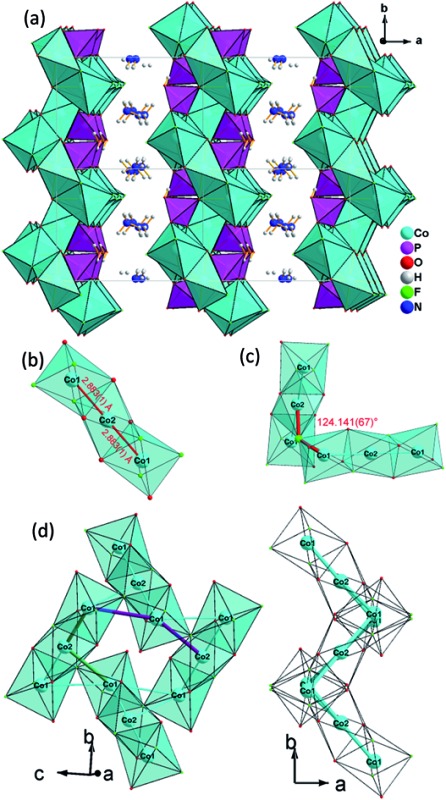
Four open-framework transition-metal phosphates; (NH_4_)_2_Co_3_(HPO_4_)_2_F_4_ (**1**), (NH_4_)Co_3_(HPO_4_)_2_(H_2_PO_4_)F_2_ (**2**), KCo_3_(HPO_4_)_2_(H_2_PO_4_)F_2_ (**3**), and KFe_3_(HPO_4_)_2_(H_2_PO_4_)F_2_ (**4**); are prepared by ionothermal synthesis using pyridinium hexafluorophosphate as the ionic liquid.

## Introduction

Inorganic open-framework materials such as molecular sieves are extensively used in many industrial processes such as heterogeneous catalysis, adsorption, ion-exchange, and separation,^1^ and the preparation of new inorganic open-framework materials is one of the most vibrant fields in materials science. Recently, the exploration of new types of open-framework material such as metal phosphates and metal borophosphates with rich structural varieties has emerged.^2,3^ Besides their traditional applications in catalysis, adsorption and separation based on the porosity in the structures, these new materials have shown great promise for applications in optics, electronics and magnetism.

In the field of open-framework metallophosphates, cobalt phosphates are of particular interest as Co^2+^ is one of the few transition metal cations that can accommodate in a tetrahedral coordination environment and thus is able to substitute silicon or phosphorus in silicates and phosphates. The incorporation of cobalt cations into zeolites can improve the catalytic performance of the material.^4^ In addition, cobalt phosphates are also of interest because of their magnetic properties.^5^ Since the first amino-templated open framework cobalt phosphate, [C_2_H_10_N_2_][CoPO_4_], was reported in 1994, much attention has been paid to this class of compounds.^6^ However, the transition metal iron is much more abundant than cobalt and no wonder, a large variety of natural iron phosphates is known, also featuring an enormous structural diversity.^7^ Iron phosphates have been vigorously pursued as environmentally friendly and inexpensive positive electrode materials for lithium-ion batteries since their initial report in 1997.^8^ The most prominent open-framework iron phosphate mineral is cacoxenite, [AlFe_24_(OH)_12_(PO_4_)_17_(H_2_O)_24_]·51H_2_O containing a 24-ring channel structure.^9^ Since the first synthetic iron phosphate analogues of the minerals hureaulite and alluaudite were reported in 1986,^10^ many 3-D iron phosphates have been prepared in the laboratory through hydrothermal methods.^2,11,12^ Successful syntheses of open-framework iron phosphates opened the way to a new family of open-framework materials that combine the well-known features of molecular sieves with magnetic properties.

Transition metal compounds with Kagomé topology, featuring two dimensional corner-sharing triangles have attracted significant interest because of their fascinating magnetic properties such as spin frustration, long-range antiferrimagnetism at low temperature and spin-canting.^13^ Most investigations of Kagomé topological materials have focussed on the jarosite family, AM_3_(SO_4_)_2_(OH)_6_ (A = alkali metal ion, M = Fe^3+^, Cr^3+^, V^3+^), and its analogues.^14^ To our knowledge, no cobalt and iron phosphates containing a Kagomé lattice have been reported. A variety of cobalt and iron phosphates with various dimensionalities and compositions have been prepared using hydro- and solvothermal methods, with or without organic amines as templates. However, until now little attention has been paid to the ionothermal syntheses of metal phosphates. In 2004, Morris coined the term ionothermal synthesis when he applied ionic liquids in the synthesis of open-framework aluminum phosphates.^15^ Ionothermal synthesis is currently receiving great interest because of the variability and tuneability of the chemical and physicochemical solvent properties that ionic liquids allow compared to traditional solvents such as water or classical volatile organic solvents;^16^ indeed, many new open-framework compounds, such as metal-, boro-, alumino- and silicoaluminophosphates and organic–inorganic hybrid materials with interesting structural architectures, new compositions, and attractive properties have been prepared recently by ionothermal synthesis.^17^ Moreover, the ionothermal synthesis of an inorganic–organic hybrid [NH_4_]_2_[C_7_H_14_N][V_7_O_6_F_18_], containing an S = 1/2 Kagomé network, showing a high degree of magnetic frustration, was reported.^18^

Hydrogen fluoride is traditionally used in the synthesis of open-framework structures as it is one of the best mineralizers.^19^ However, particularly when large amounts of F^–^ ions are employed, fluoride itself is often incorporated in the reaction product. This further broadens the structural variety as fluoride can not only bridge transition metal cations but also act as a terminal site in (fluoro)phosphate polyhedra. Recently, Weller's group successful synthesized about 50 transition metal (Mn to Cu) framework fluorophosphates in fluoride-rich hydrothermal media from metal fluorides.^20^ Meanwhile, fluoride-based materials have regained interest in the field of Li-ion batteries.^21^ Several fluorophosphate materials have been studied as electrodes such as Na_2_FePO_4_F,^22^ Li_5_V(PO_4_)_2_F_2_,^23^ LiVPO_4_F,^24^ and LiMPO_4_F (M = Fe, Ti, Co, Cr).^25^ Recham *et al.* used ionothermal methods in the successful low-temperature synthesis of LiMPO_4_F (M = Ti, Fe),^26^ Na_2_MPO_4_F (M = Fe, Mn),^27^ Na_2_Fe_1–*x*_Mn_x_PO_4_F^28^ and LiFeSO_4_F^29^ for Li-ion battery applications.

Recently, our group has successfully utilized ionic liquids in the synthesis of metal, and metal fluoride as well as oxide nano-materials for energy-related applications such as in catalysis or photonic materials.^30^ Simultaneously, we explored a wide range of different IL cations and anions in the synthesis of layered and framework alumophosphates,^31^ cobalt hydroxyl phosphate and borophosphates.^32,33^

To explore the synthesis of new magnetic transition metal materials from ionic liquids, we have attempted the synthesis of cobalt/iron fluorophophates using pyridinium hexa-fluorophosphates as both solvent and fluoride source. Herein, we report the synthesis and characterization of one layered cobalt fluorophosphate (NH_4_)_2_Co_3_(HPO_4_)_2_F_4_ (**1**) and three isostructural open-framework cobalt/iron phosphates (NH_4_)Co_3_(HPO_4_)_2_(H_2_PO_4_)F_2_ (**2**), KCo_3_(HPO_4_)_2_(H_2_PO_4_)F_2_ (**3**), and KFe_3_(HPO_4_)_2_(H_2_PO_4_)F_2_ (**4**), using the ionic liquid, 1-butylpyridinium hexafluorophosphate, [C_4_py][PF_6_] or 1-butyl-4-methylpyridinium hexafluorophosphate, [C_4_mpy][PF_6_]. Their structures and magnetic properties are investigated in detail.

## Results and discussion

### Synthesis

Compounds **1–4** could be obtained phase pure (for PXRD data see ESI†) by reaction of the respective transition metal halide or acetate with a hydrogenphosphate in a pyridinium hexafluorophosphate ionic liquid. The 2D-layer compound **1** and 3D compound **2** could be obtained in the same ionic liquid **[C_4_py][PF_6_]**, with the same reactants under the same conditions, simply by controlling the phosphate to cobalt ratio. Compounds **3** and **4**, isostructural to **2** could be obtained in the ionic liquid **[C_4_mpy][PF_6_**]. Here, the phosphate source was varied from the ammonium to the potassium salt, with compound 4 based on Fe instead of Co. Compounds bearing structural similarities to compounds **2–4** have previously been obtained hydrothermally using CoF_3_, H_3_PO_4_, and NH_4_OH/KOH.^17a,b^ As seen in the synthesis of compounds **1** and **2**, a careful choice of the reaction parameters is mandatory to obtain phase-pure materials. On careful examination of the influencing factors, we found that the molar ratio of the starting materials is a critical factor in the formation of compounds **1** and **2**. Pure compound **1** can be made by treating 2 equiv. of Co(CH_3_COO)_2_·4H_2_O, 1 equiv. of H_3_BO_3_, 1 equiv. of NH_4_H_2_PO_4_ and 2 equiv. of [C_4_py][PF_6_] and at 200 °C for 5 days. When the P/Co ratio is increased to 1, large single crystals of phase-pure compounds **2** are obtained. When the P/Co ratio lies between 1/2 and 1, compounds **1** and **2** coexist in the product. As the ratio increases, compound **2** is the predominating phase.

### Structures

#### (NH_4_)_2_Co_3_(HPO_4_)_2_F_4_ (**1**)

**Fig. 1:**
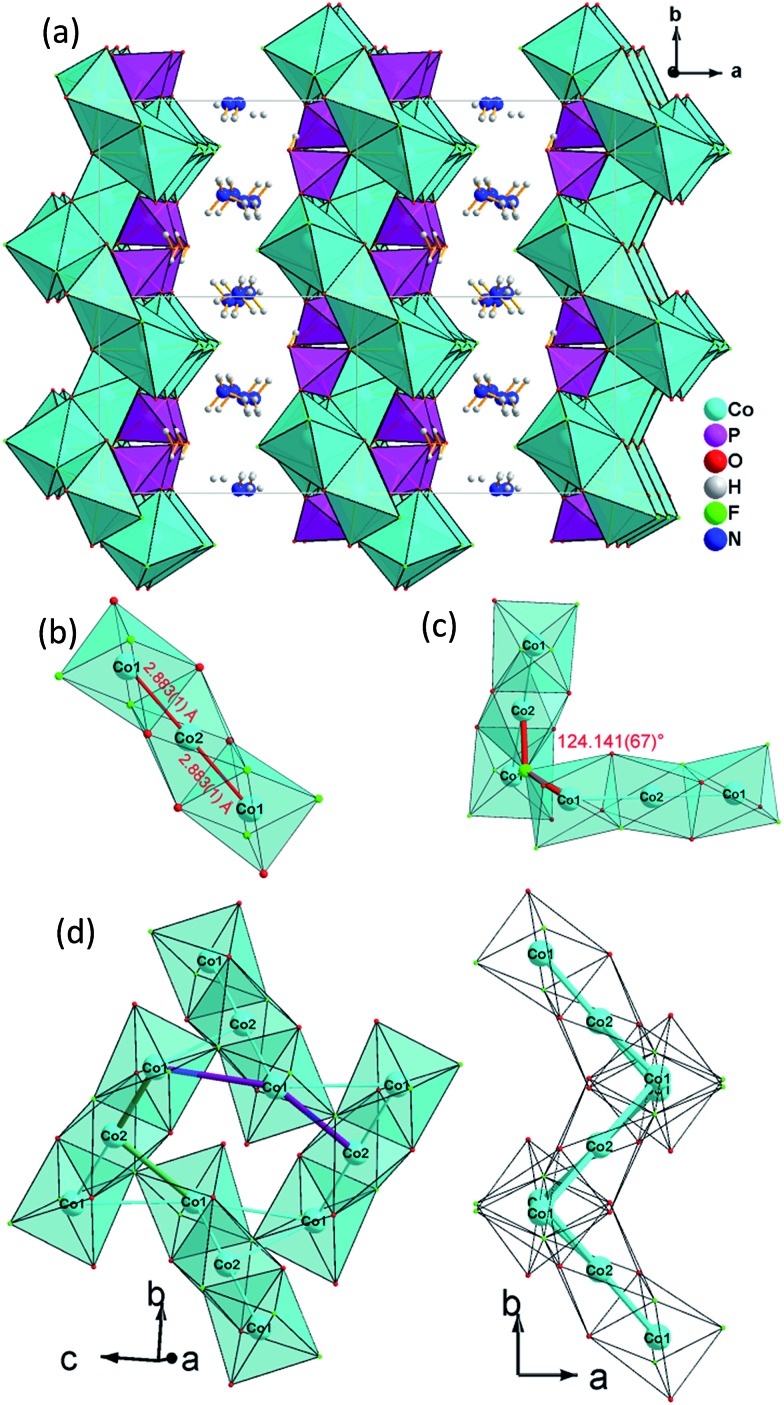
(a) View of the layered structure of (NH_4_)_2_Co_3_(HPO_4_)_2_F_4_ **(1)** along the [001] direction; (b) trimeric [Co_3_O_6_F_6_], octahedra basic building unit (BBU); (c) dimeric triple [Co_3_O_6_F_6_] octahedra as the fundamental building unit (FBU) (c) 3,6-ring cobalt fluoroxide octahedra unit composed by two FBUs viewed along [100] (left) and [001] (right).

Single-crystal X-ray diffraction analysis reveals that (NH_4_)_2_Co_3_(HPO_4_)_2_F_4_ (**1**) obtained from 1-butyl-pyridinium hexafluorophosphate crystallizes in the monoclinic space group *P*2_1_/*c*. Its structure consists of {Co(HPO_4_)F_4_}, macroanionic layers and charge compensation being achieved by NH_4_^+^ ions (Fig. 1a). The asymmetric unit contains 10 non-hydrogen atoms, as shown in Fig. S4a,† with two crystallographically independent Co atoms and F atoms and one crystallographically independent P atom and N atom.

All atoms lie on the general position except for Co(2), which is found at a special position (2a). Nevertheless both cobalt ions feature a distorted octahedral coordination environment: Co(1) is surrounded by two μ3-F, two μ3-O and one μ-O atom to form four Co–F–Co, one Co–O–Co and two Co–O–P bonds, leaving one terminal F, exhibiting the shortest Co(1)–F(2) distance (2.0014(17)Å) (Table 2 and ESI†). Co(2) lies at the origin and is coordinated by two μ3-F and four μ3-O atoms to form four Co–F–Co, Co–O–Co and Co–O–P bonds, respectively. The Co–O, Co–F bond lengths are in the range of 2.0461(19)–2.121(2) Å and 2.0014(17)–2.1448(15) Å, respectively. The phosphorus atom is tetrahedrally coordinated by oxygen atoms and is connected *via* two μ3-O and one μ-O atom to nearby Co atoms, leaving one terminal P–O(4)H bond with larger bond length (1.588(5) Å). The P–O bond lengths vary in the range of 1.507(5)–1.527(5) Å, which is in accordance with the reported values. The N(1)H_4_^+^ group interacts with the terminal atom F(2) of the [CoO_3_F_3_] octhedron *via* a hydrogen bond; the N–H/F distances are in the range of 2.660(3)–3.091(3) Å, and with the terminal atom O(4) and the bridging oxygen O(3) atoms of the phosphate group, the N–H/O distances are in the range of 2.911(3)–3.049(3) Å.

The 2D layer structure of (NH_4_)_2_Co_3_(HPO_4_)_2_F_4_ (1) arises from connection of [CoO_3_F_3_], [CoO_4_F_2_] octahedra and [PO_3_OH] tetrahedra (Fig. 1a). The most important structural feature is a cobalt fluoroxide octahedra trimer, [Co_3_O_6_F_6_], which is formed by connection of two [Co(1)O_3_F_3_] octahedra to the opposite faces of a central [Co(2)O_4_F_2_] octahedron. The Co(1)–Co(2) distance is 2.883(1) Å (Fig. 1b). Two of these triple octahedral units are further connected by sharing a fluoride ion between the terminal [Co(1)O_3_F_3_] octahedra at an angle Co(1)–F(1)–Co(2) of 124.141(67)° (Fig. 1c).

The resulting Co(1)–Co(1) distance is 3.809(1) Å. This results in the formation of corrugated layers with Kagomé topology featuring 3,6-rings with angles of 110.820(9)° (Co(1)–Co(2)–Co(1)) and 107.270(11)° (Co(1)–Co(1)–Co(2) (Fig. 1d and 3a). These layers are stacked along the *a* axis in a sequence AA… (Fig. 3b). Hydrogen phosphate groups augment the transition metal layer on both sides, leaving one terminal OH group.

### (NH_4_)Co_3_(HPO_4_)2(H_2_PO_4_)F_2_ (**2**), KCo_3_(HPO_4_)_2_(H_2_PO_4_)F_2_ (**3**), KFe_3_(HPO_4_)_2_(H_2_PO_4_)F_2_ (**4**)

**Fig. 2:**
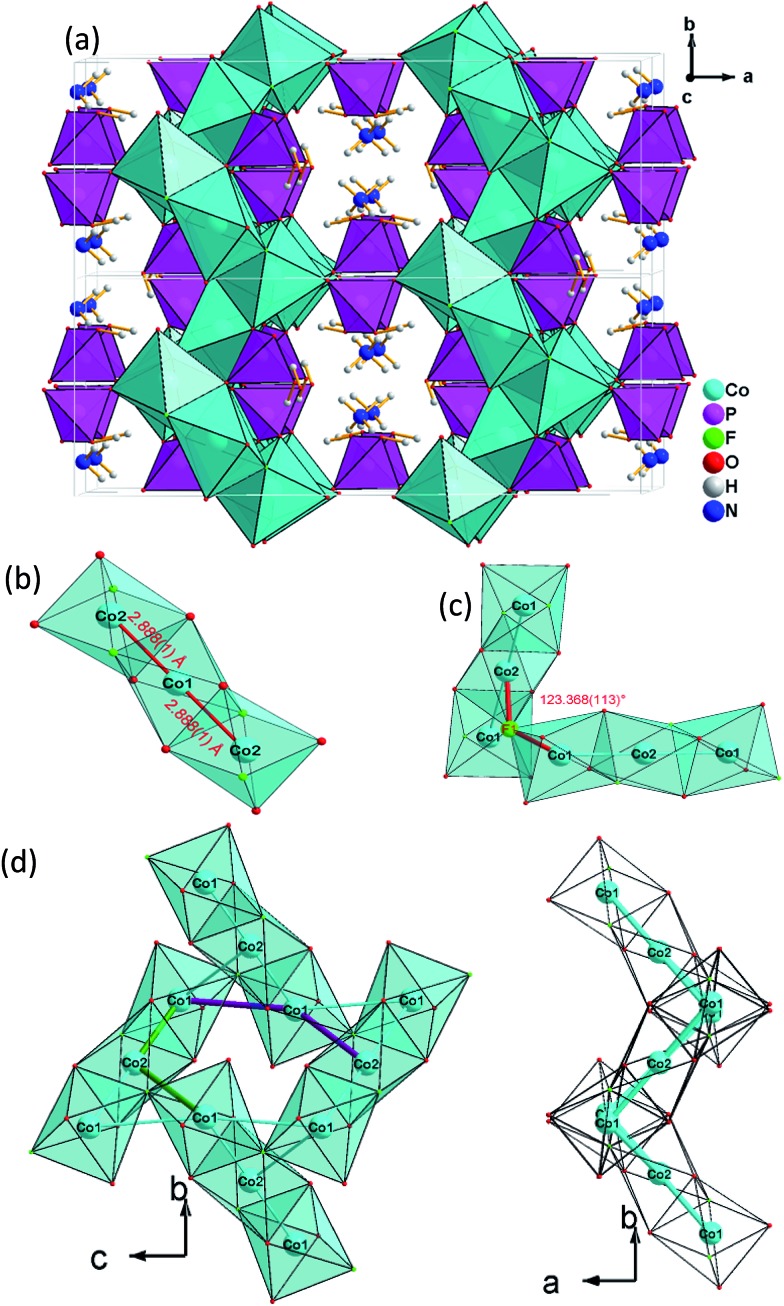
(a) View of the 3D open-framework structure of (NH_4_)Co_3_(HPO_4_)_2_(H_2_PO_4_)F_2_ **(2)** along the [001] direction showing 10-ring channel; (b) trimeric [Co_3_O_8_F_4_] octahedra basic building unit (BBU); (c) dimeric triple [Co_3_O_8_F_4_] octahedra as the fundamental building unit (FBU) (c) 3,6-ring cobalt fluoroxide octahedra composed by two FBUs viewed along [100] (left) and [001] (right).

Single-crystal XRD analyses show that the compounds **2–4**, obtained from pyridinium hexafluorophosphate ionic liquids crystallize isostructurally in the space group *C*2/*c* and are 3D framework structures consisting of a {M(HPO_4_)_2_(H_2_PO_4_)F_2_} macroanionic framework where charge compensation is achieved either by K^+^ or NH_4_^+^ ions (Fig. 2a). The asymmetric unit of (NH_4_)Co_3_(HPO_4_)2(H_2_PO_4_)F_2_ (**2**), which will be discussed as a representative of the set of isostructural compounds **2–4**, contains two crystallographically distinct P atoms and Co atoms, one crystallographically distinct N atom and one F atom (Fig. ESI-4b†). The structure features two different phosphate units: a hydrogenphosphate group, [P(1)O_3_OH], and a dihydrogenphosphate group, [P(2)O_2_(OH)_2_].

The interatomic distances in the P–O bridging atoms are in the range of 1.491(3)–1.517(3) Å, and for the protonated, terminal P–OH atoms as expected slightly longer with values of 1.565(3) Å (P(1)–O(6)) and 1.569(3) Å (P(2)–O(4)). These data are in good agreement with the literature values for phosphate and hydrogenphosphate units. Similar to compound **1**, the two Co atoms also feature a distorted octahedral environment. However, both cobalt ions are surrounded by four oxide and two trans-fluoride anions, whereas in **1** both a [CoO_3_F_3_] unit and a [CoO_4_F_2_] octahedron are found. The [Co(2)O_4_F_2_] octahedron is connected *via* opposite faces to two [Co(1)O_4_F_2_] octahedra forming an octahedron trimer (BBU) (Fig. 2b), similar to **1**. The Co–O bond lengths vary in the range of 2.026(3)–2.125(3) Å (Table 2 and ESI†). Co–F bond lengths are found between 2.082(2) and 2.187(2) Å. Similar to **1**, these octahedral trimers are connected to each other *via* μ3-F atoms at an Co(1)–F(1)–Co(2) angle of 123.368(113)° (Fig. 2c) resulting in a corrugated layer containing 3,6-rings with Kagomé topology parallel to the *bc* plane (Fig. 2d, 4a). The Co(1)–Co(2) distance is 2.888(1) Å and the Co(1)–Co(1) distance is 3.809(1) Å. In contrast to **1** the Kagomé layers are stacked along the *a* axis in a sequence AA_*i*_…, where the layers A and A_*i*_ are related by inversion symmetry (i) (Fig. 4b).

**Fig. 3:**
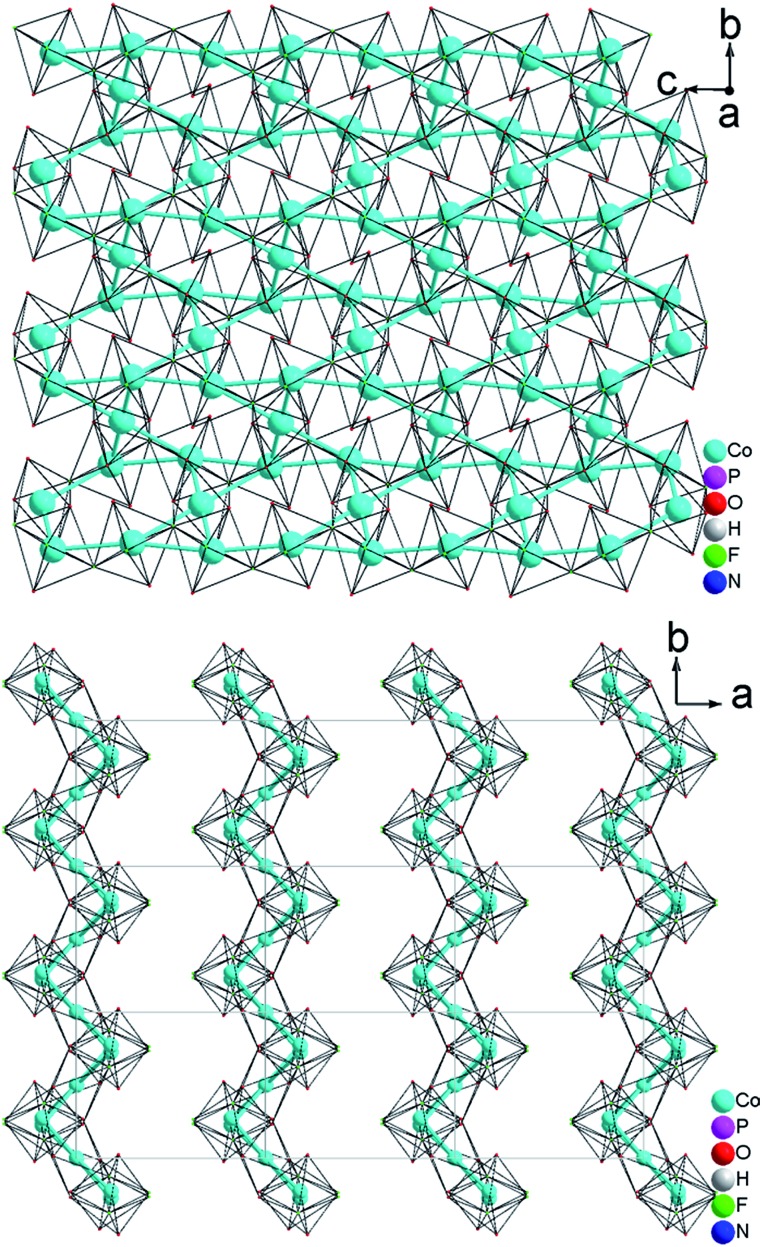
(a) 2-D [MO_4_F_2_]_*n*_^8*n*–^ Kagomé net (NH_4_)_2_Co_3_(HPO_4_)_2_F_4_ (**1**) parallel to the *bc* plane; (b) a viewing showing that the 2D metal—Kagomé layer stacked along *a* axis in a sequence AA… in (NH_4_)_2_Co_3_(HPO_4_)_2_F_4_ (**1**).

**Fig. 4:**
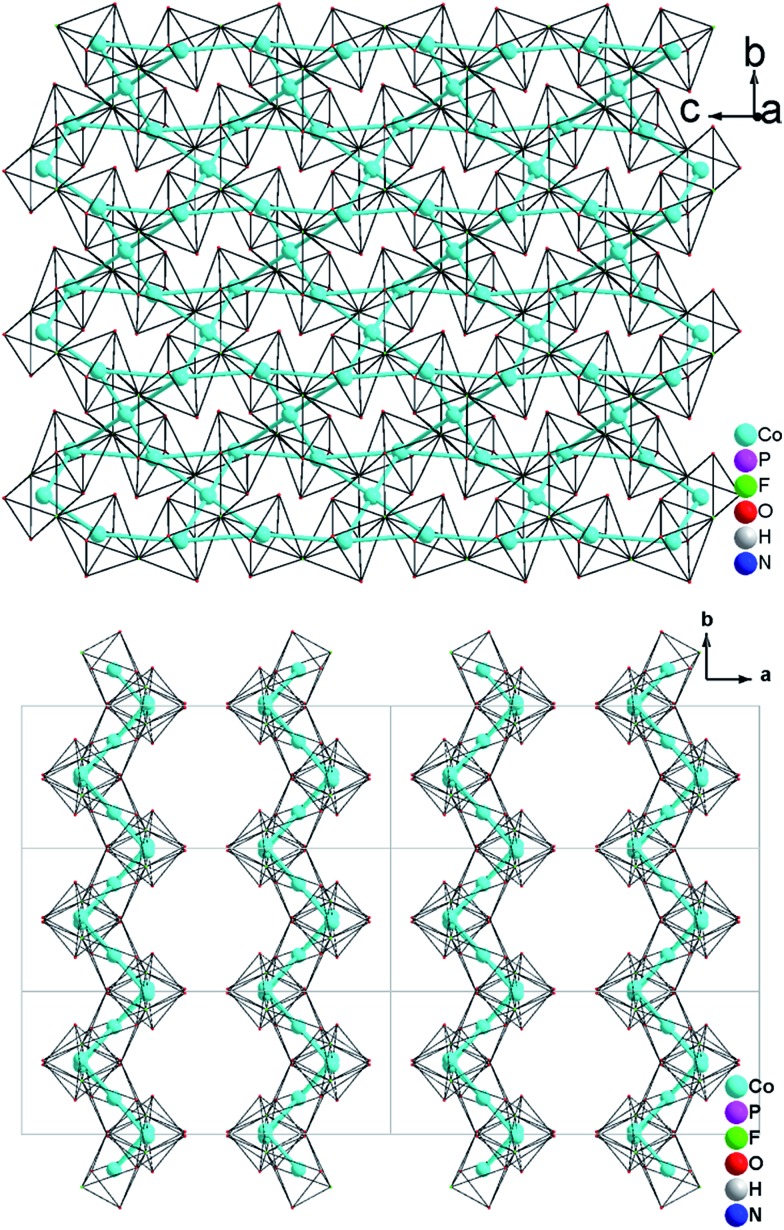
(a) 2-D [MO_4_F_2_]_*n*_^8*n*–^ Kagomé net in (NH_4_)Co_3_(HPO_4_)_2_(H_2_PO_4_)F_4_ (**2**) parallel to the *bc* plane (b) A viewing showing that the 2D metal—Kagomé layer stacked along *a* axis in a sequence AA_*i*_… in (NH_4_)Co_3_(HPO_4_)_2_(H_2_PO_4_)F_4_ (**2**).

Similar to **1**, phosphate tetrahedra (P(1)O_3_OH) augment the Co–O–F Kagomé topology layer on both sides leading to a composition for the layer of Co_3_O_2_(HPO_4_). These layers are interlinked by P(2)O_2_(OH)_2_ groups to form a 3-D open-framework structure, forming 10-ring channels along the [001] direction, in which NH_4_^+^ ions are located (Fig. 2a).

### IR spectroscopy

IR spectra for compounds **1–4** recorded in the region 4000–400 cm^–1^ were collected to confirm the presence of P–O–H and N–H moieties (Fig. S2, ESI†). The broad band observed around 3220 cm^–1^ can be attributed to the stretching vibration of hydroxyl groups of the phosphate tetrahedra. The two weak bands at about 2450 and 2140 cm^–1^ are due to *μ*(P–OH) and the overtone *δ*(P–O–H), respectively and are very characteristic of the hydrogen phosphate groups. The bands at 1420 cm^–1^ and 3310 cm^–1^ which can be observed in compounds **1** and **2**, originate from vibrations of the NH_4_^+^ group.

### Thermal analysis

The thermal behavior of all reported materials was investigated using thermogravimetric (TG) analysis under N_2_ flow (Fig. S3†).

The TGA curve of compound **1** (green line) shows two stages of weight loss occurring from 300 to 600 °C. The first weight loss of a total of 15.0 wt% (calc.: 15.0 wt%) in the region of 300–400 °C corresponds to the loss of the ammonium cation between the layers. The second of 6.89 wt% (calc.: 7.1 wt%) from 530 to 570 °C is ascribed to the removal of OH groups by dehydration and the loss of the fluoride anion. The TG curves of compounds **2–4** show that all three are stable up to about 450 °C, and then undergo a one-step weight loss of from 450 °C to 550 °C, corresponding to the release of H_2_O/NH_3_ and HF. (NH_4_)Co_3_(HPO_4_)_2_(H_2_PO_4_)F_2_ (**2**) (red line), shows a large weight loss of 17.9 wt% (calc., 16.09 wt%) which can be attributed to the elimination of the ammonium cation, the dehydration of the terminal P–OH groups and the loss of the fluorine anion. The isostructural potassium compounds, KCo_3_(HPO_4_)_2_(H_2_PO_4_)F_2_ (**3**) (black line) and KFe_3_(HPO_4_)_2_(H_2_PO_4_)F_2_ (**4**) (blue line) show a similar thermal behavior with a total weight loss of about 10.63 wt% (calc., 10.68 wt%) and 11.46 wt% (calc., 12.7 wt%) in the region 400–550 °C corresponding to dehydration of the terminal P–OH groups and the loss of the fluoride anion. X-ray powder diffraction confirm that the structures of **1–4** collapse upon heating, when heating samples of **1–4** to 800 °C, mixtures of dense phases Co/Fe_2_(P_2_O_7_) and Fe_3_(PO_4_)O_3_/Co_3_PO_4_ are formed.

### Magnetism

**Fig. 5:**
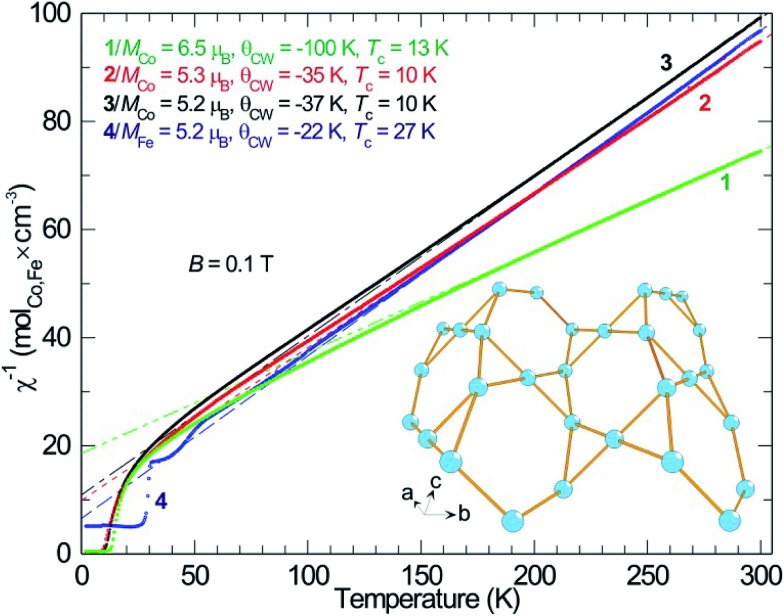
Reciprocal magnetic DC-susceptibility (*χ* ^–1^) plotted *versus* temperature for compounds **1–4**, together with the data obtained from the respective Curie–Weiss fit (dashed lines) and the observed magnetic transition temperatures. The lower right inset shows the general transition metal sublattice of compounds **1–4** to illustrate the structural analogy to a stair-case Kagomé net.

**Fig. 6:**
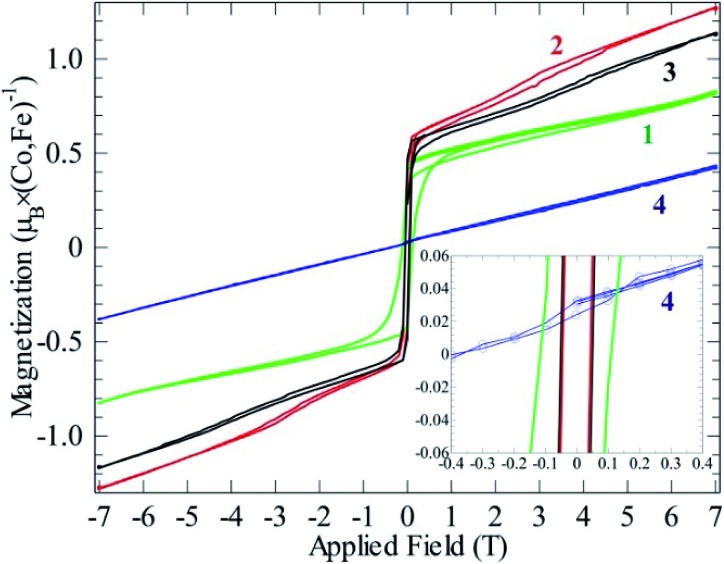
Magnetizations at 2 K as functions of field for compounds **1**–**4**. All curves contain a full hysteresis loop.

**Fig. 7:**
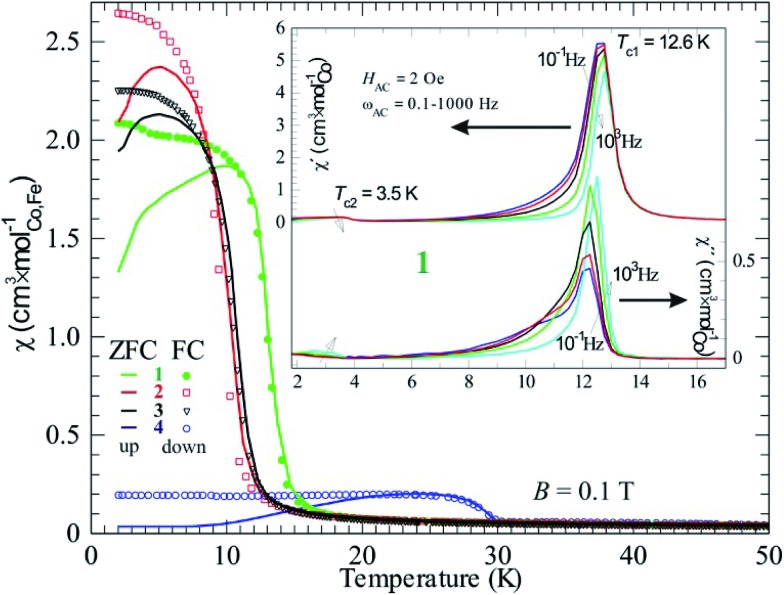
Magnetic DC-susceptibility plotted *versus* temperature for compounds **1–4**. All zero-field cooled (ZFC, full lines) curves were measured on heating (up) but the field-cooled (FC, individual markers) curves were obtained on cooling. The inset displays the AC-susceptibility of compound **1** with both real part (*χ*′, left ordinate axis) and imaginary part (*χ*′′, right axis). The different curves represent different frequencies (0.1, 1, 10, 100, 1000 Hz) of the 2 Oe driving field.

As described, all four compounds **1–4** contain a transition metal sublattice that is built up by layers which resemble a puckered (“stair-case”) Kagomé network (see Fig. 5 & 6 and inset in Fig. 7). Kagomé lattices feature corner-sharing triangles,^34^ promising for magnetic frustration.^35^ It is expected from the structures of compounds **1–4** that antiferromagnetic nearest neighbor spin interactions (AF) for the transition metal cations occur *via* superexchange through O^2–^ and F^–^ that should cause geometric frustration.

Investigating the magnetic properties of compounds **1–4** in detail reveals that the high temperature magnetic behavior of the four compounds is similar: Curie–Weiss paramagnetism with preference for antiparallel spin–spin couplings (Fig. 5, negative Weiss-constant, *θ*_CW_). From the Curie–Weiss fit, the effective moments (*μ*_eff_) of Co^2+^ are estimated to be 6.5 *μ*_B_ (compound **1**), 5.3 *μ*_B_ (compound **2**), 5.2 *μ*_B_ (compound **3**) and, whereas the moment for Fe^2+^ (compound **4**) is similar in size, 5.2 *μ*_B_ (Fig. 6). A d^7^ ion such as Co^2+^, in octahedral environment and in the high-spin (hs) state is generally described as a *S* = 3/2 entity, corresponding to an effective moment of 3.87 *μ*_B_. Obviously, this does not match with the relatively large magnetic moments found for the Co compounds. Instead, full orbital moments must be considered to account for the observations, *i.e.* for Co^2+^ ions, *S* = 3/2 and *L* = 3 (*J* = 9/2), resulting in a theoretical effective moment of 5.20 *μ*_B_.

This fits well to the moment of Co^2+^ in compounds **2** and **3**, however, compound **1** exhibits an even higher moment, and at the same time about three times stronger antiferromagnetic (AF) coupling (*θ*_CW_ = –100 K). This probably means that the true paramagnetic state has not been reached at 300 K for compound **1** and the Curie–Weiss fit does not supply proper values. For the iron compound **4** which is isostructural to compounds **2** and **3** containing hs-d^6^ Fe^2+^ (*S* = 2) the full orbital moment is also needed to describe the measured effective moment; S + L corresponds in this case to 5.48 *μ*_B_ compared to the spin-only (S) value of 4.90 *μ*_B_. Orbital contributions to the magnetic moment are common for Co^2+^, but both its size and the fact that Fe^2+^ shows a similar behavior should be investigated further in the future. Although *θ*_CW_ is negative for all compounds, an abrupt increase in magnetic susceptibilities (*χ*) marks spin ordering with ferromagnetic components close to 10 K for both cobalt compounds **2** and **3**, while compound **1** orders at 13 K and the iron spins in **4** order at 27 K. Possible magnetic scenarios are either a ferrimagnetic ground state with an uneven distribution of antiparallel spins or with spin canting away from an ideal antiferromagnetic (AF) state. The spins in a ferrimagnet must be flipped completely (180°) in a magnetization measurement in contrast to the flopping of spins in a canted AF (≪180°). The remnant field in the former scenario case often is larger than in the latter. Hence, the magnetic ordering of spins in **1**, and **2–3** most likely can be described as canted AFs, as the remnant field is relatively small (Fig. 6); for a simple ferrimagnet at least one full spin must remain uncompensated at fields beyond the hysteresis, *i.e.* 1 *μ*_B_ per spin-only Co (3 *μ*_B_ per formula unit) or 3 *μ*_B_ per Co (9 *μ*_B_ per formula unit) if the orbital moment is as active as observed in the paramagnetic state. Completely different behavior is seen for **4** as only a linear increase in magnetic polarization is observed as function of applied field. The linear increase in magnetization with rising magnetic field is a typical observation for canting out of a canted- or normal-AF state *i.e.* the energy of the B-field is working towards full spin polarization. The sudden change in magnetization for the Co containing compounds is probably due to a spin-flop process where the canting can occur in direction of an easy axis or in the opposite direction.

Compound **1** exhibits significant hysteresis with a coercive field of 0.1 T (inset Fig. 6), again proving the relatively stronger magnetic coupling in the system as expected from the by far largest value of *θ*_CW_. Both compounds **2** and **3** only exhibit half of the coercive field of compound **4** (50 mT). The reason for compound **4** not having a spin-flop within the measured field range can be understood by a close examination of the magnetization curve. In fact, compound **4** does not cross zero magnetization at *B* = 0 but has a constant remnant magnetization that needs an opposite field of 0.4 T to be compensated (inset Fig. 6). A possible reason for this is that the easy axis for Fe^2+^ cannot flop to the anti-parallel direction through a local non-centrosymmetry or dependent on the different electron configuration d^6^ of Fe^2+^ in comparison to d^7^ of Co^2+^. This forces the spin to cant in one specific way in the absence of an outer field. By applying a field the spin can either cant further along this direction or into a meta-stable AF state at –0.4 T that can then be further distorted on increasing the field. Hence, Fe^2+^ seems to possess a stronger spin orbit coupling compared to Co^2+^ in these compounds. Normally, a powder, where all crystallographic directions are equally abundant, would not exhibit this offset, which means that we have minor texture effects in the data of compound **4**. However, without the texture effect it would not have been possible to extract this information from the available data. By simple deduction, it is possible to calculate the approximate canting angle from the jump in the magnetization (*μ*_jump_) of **1** (0.5 *μ*_B_) and **2**–**3** (0.6 *μ*_B_) and the expected saturation moment (*μ*_sat._) for **1** (5.04 *μ*_B_), **2** (4.11 *μ*_B_), and **3** (4.03 *μ*_B_). These saturation moments were calculated by extracting the Landé factor (*g*) from the paramagnetic range using 
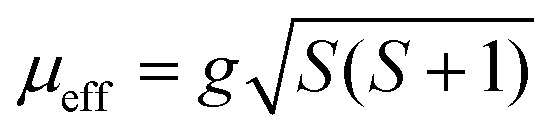
 and inserting it in the simpler formula for an ordered moment: *μ*_sat._ = *gS*. The canting angle (*Φ*) can then be extracted by = sin^–1^(*μ*_jump_/*μ*_sat._), giving 6°, 8°, and 9° for **1**–**3**, respectively.

By comparing susceptibility data from field cooled (FC) and zero-field cooled (ZFC) samples it is obvious that all four compounds form magnetic domain structures, as FC results in a higher *χ* than ZFC (Fig. 7). This is normal for magnetic ordering with a ferromagnetic component, due to the fact that the magnetic transition is of second order and the absence of a field results in a domain structure without orientational preference. Macroscopically this state is called superparamagnetism, a state that also show frequency dependence when applying an AC-field. This is obviously the case, as the expected behavior is seen for example in compound **1** (inset Fig. 7). The observed *T*_c_ in the real part of *χ* (*χ*′) shifts towards higher temperature on increasing the AC driving frequency (*ω*_AC_), which is depicted with an arrow in Fig. 7; the same is valid for the imaginary part of *χ* (*χ*′′). Even the relatively large imaginary part suits a superparamagnetic state, where 5–10% magnetic energy loss (*χ*′′) is observed. This means that the domains are relatively large and, thus, slow. Also, fitting into the suggested picture is the decreasing (increasing) *χ*′ (*χ*′′) on increasing *ω*_AC_. The magnetic domain structure is naturally only observed at lower fields (within the hysteresis) and higher fields, *e.g.* above 1 T, would force ZFC data to superimpose FC data.

It should be noted that a second transition is obvious in the AC data at about 3.5 K. This agrees with the DC data, where a counterpart is seen as a kink in both FC and ZFC curves for compound **1**. On lowering the temperature, more spin interactions come into play and the minor changes in *χ* most probably originate from a spin reorientation. Its nature can be further understood by using neutron diffraction.

It should be mentioned that there is a significant temperature hysteresis between the FC and ZFC curves, *i.e.* down and up curves, respectively. For pure magnetic transitions there should not be any hysteretic effects unless the heating/cooling rate is too high and causes temperature inhomogeneities, meaning that the sample has a different temperature than its surroundings. Here, the rate was chosen to be low and the hysteresis must be due to another effect. A possible explanation is that the phase transition has an intermixing of first order character. This suggests that a structural transition can be coupled to the magnetic ordering event, often presented as a magnetoelastic coupling. Naturally, the magnetoelasticity must be further proven, however there are no further data at hand.

**Fig. 8:**
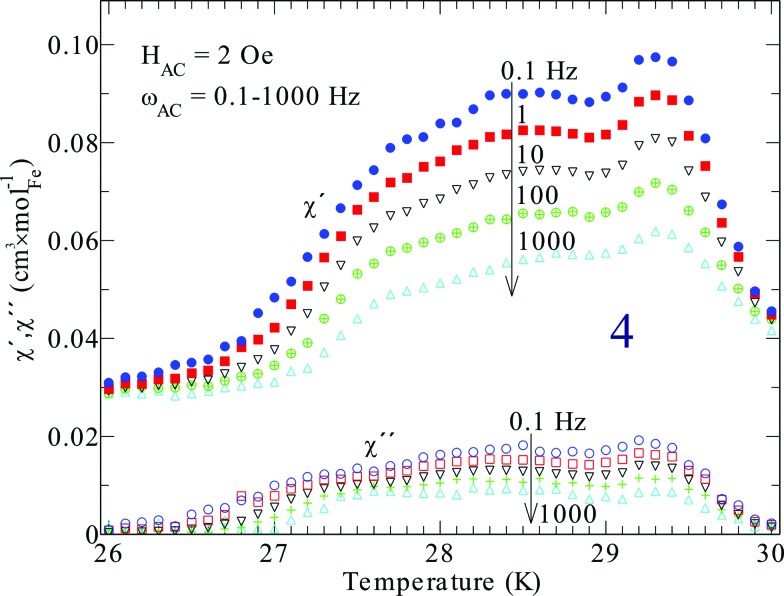
Alternating field magnetic susceptibility of compound **4** at different driving frequencies, as indicated in the inset. Note, both real (χ′) and imaginary part (χ′′) have the same ordinate axis and the arrows indicate increasing frequency.

Compound **4** contains iron and has a significantly higher magnetic transition temperature and behaves different in DC susceptibility data (Fig. 7) and magnetization (Fig. 6). Hence, the transition temperature of **4** was further investigated by AC-susceptibility (Fig. 8) 15–20% of the signal, measured at 1000–0.1 Hz, is magnetic energy loss (χ′′) indicating that relatively large spin domains are formed on cooling through 30 K. Further, the loss (χ′′) stays almost relative to the observed signal (χ′), only increasing slightly on decreasing the measuring frequency. This would mean that the true resonance with the spin system has not been reached even at 0.1 Hz. This suggests that even slower spin fluctuations dominate in the measured temperature range, agreeing with the proposed relatively large spin domains. Moreover, the spin freezing progresses in several steps and is completed at about 26 K. This behavior is somewhat unexpected and needs further investigations, perhaps with Mössbauer spectroscopy or myon spin resonance measurements.

Antiferromagnetically coupled spins on a triangular lattice with only nearest neighbor spin interaction often leads to geometric frustration, which can be roughly quantified by calculating the ratio *θ*_CW_/*T*_c_ (or *T*_N_). In all compounds, expect for perhaps compound **1**, this value is low and the magnetic systems somehow seem to overcome the frustration. A probable explanation is that next nearest neighbor spin interactions, especially between the Kagomé layers, are important and the geometric frustration becomes negligible.

The magnetic complexity of stair-case Kagomé systems is illustrated by the transition metal vanadates, Me_3_V_2_O_8_ (Me = Cu, Co, and Ni).^36^ The highly symmetric orthorhombic space group *Cmca*, in which those vanadates crystallize, restricts the magnetic properties to an AF structure. However, the monoclinic symmetry of the title compounds **1–4** allows for more spin freedom, which might be the reason for spin canting in the AF ground state.

In their ground states, all four compounds exhibit long range magnetic order and can be described as canted anti-ferromagnets.

## Experimental

### Synthesis

(NH_4_)_2_Co_3_(HPO_4_)_2_F_4_ (**1**), (NH_4_)Co_3_(HPO_4_)_2_(H_2_PO_4_)F_2_ (**2**), KCo_3_(HPO_4_)_2_(H_2_PO_4_)F_2_ (**3**), and KFe_3_(HPO_4_)_2_(H_2_PO_4_)F_2_ (**4**) were prepared under ionothermal reaction conditions using the ionic liquid 1-butylpyridinium hexafluorophosphate, [C_4_py][PF_6_] (98%, Merck) (**1–2**) or 1-butyl-4-methylpyridinium hexafluorophosphate, [C_4_mpy][PF_6_] (99%, Merck) (**3–4**) as both solvent and fluoride source. In a typical reaction the appropriate amounts of the transition metal salt and a hydrogen- or dihydrogenphosphate as the phosphate source were dissolved in the chosen ionic liquid. The reaction mixtures were reacted in a 3 ml/50 ml Teflon-lined stainless steel reaction vessel for 5–7 days at 180–200 °C. After cooling to room temperature, the products were filtered off, washed with deionized water and acetone, and dried at 60 °C for one day. Phase purity was confirmed by the agreement between the experimental powder X-ray diffraction (XRD) patterns and the simulated patterns based on the structure analysis (Fig. S1†).

(NH_4_)_2_Co_3_(HPO_4_)_2_F_4_ (**1**): 0.5018 g Co(CH_3_COO)_2_·4H_2_O (98%, ACROS), 0.1942g NH_4_H_2_PO_4_ (98%, Merck) and 0.0365 g H_3_BO_3_ (99.8%, Appl. Chem.) in 0.5018 g [C_4_py][PF_6_], 200 °C, 5 days, yield: 60.5% based on Co(CH_3_COO)_2_·4H_2_O.

(NH_4_)Co_3_(HPO_4_)_2_(H_2_PO_4_)F_2_ (**2**): 0.2037 g Co(CH_3_COO)_2_·4H_2_O (98%, ACROS), 0.2037 g NH_4_H_2_PO_4_ (98%, Merck) and 0.0355 g H_3_BO_3_ (99.8%, Appl. Chem.) in 0.5023 g [C_4_py][PF_6_]. 200 °C, 5 days, yield: 69.2% based on Co(CH_3_COO)_2_·4H_2_O.

KCo_3_(HPO_4_)_2_(H_2_PO_4_)F_2_ (**3**): 0.476 g CoCl_2_·6H_2_O (98%, Fluka), 0.272 g KH_2_PO_4_ (99%, J. T. Baker) in 1.18 g [C_4_mpy][PF_6_]. 200 °C, 5 days, yield: 70% CoCl_2_·6H_2_O.

KFe_3_(HPO_4_)_2_(H_2_PO_4_)F_2_ (**4**): 0.215 g FeBr_2_ (98%, Aldrich), 0.271 g KH_2_PO_4_ (99%, J. T. Baker) and 0.03 g H_3_BO_3_ (99.8%, Appl. Chem.) in 0.509 g [C_4_mpy][PF_6_], 180 °C, 7 days, yield: 53.6% based on FeBr_2_.

### Single-crystal structure determination

Intensity data set of suitable single crystals of (NH_4_)_2_Co_3_(HPO_4_)_2_F_4_ (**1**), (NH_4_)Co_3_(HPO_4_)_2_(H_2_PO_4_)F_2_ (**2**), KCo_3_(HPO_4_)_2_(H_2_PO_4_)F_2_ (**3**), and KFe_3_(HPO_4_)_2_(H_2_PO_4_)F_2_ (**4**) were collected at ambient temperature using graphite-monochromated Mo-Kα radiation on an Image Plate Diffraction System, IPDS I, (Stoe, Darmstadt, Germany). The data were corrected for Lorentz and polarization effects. Data correction was carried out with the program X-RED.^37^ A face-indexed numerical absorption correction (X-SHAPE) was applied.^38^ The structure was solved by direct methods and refined by full-matrix least-squares techniques with the SHELXTL crystallographic software package.^39^ The Co/Fe, P, F, and O atoms could be unambiguously located. The K^+^/N^3+^ ions were subsequently located from a difference Fourier map. In the final refinement H atoms associated with the hydroxyl groups were added computationally. Experimental details on the crystal structure determination of (NH_4_)_2_Co_3_(HPO_4_)_2_F_4_ (**1**), (NH_4_)Co_3_(HPO_4_)_2_(H_2_PO_4_)F_2_ (**2**), KCo_3_(HPO_4_)_2_(H_2_PO_4_)F_2_ (**3**), and KFe_3_(HPO_4_)_2_(H_2_PO_4_)F_2_ (**4**) are listed in Table 1. Further information can be found in ESI.†

**Table 1:** Crystal data and structure refinement for (NH_4_)_2_Co_3_(HPO_4_)_2_F_4_ (**1**), (NH_4_)Co_3_(HPO_4_)_2_(H_2_PO_4_)F_2_ (**2**), KCo_3_(HPO_4_)_2_(H_2_PO_4_)F_2_ (**3**), and KFe_3_(HPO_4_)_2_(H_2_PO_4_)F_2_ (**4**)

Identification code	**1**	**2**	**3**	**4**
Empirical formula	(NH_4_)_2_Co_3_(HPO_4_)_2_F_4_	(NH_4_)Co_3_(HPO_4_)_2_(H_2_PO_4_)F_2_	KCo_3_(HPO_4_)_2_(H_2_PO_4_)F_2_	KFe_3_(HPO_4_)_2_(H_2_PO_4_)F_2_
Formula weight( g mol^–1^)	480.82	521.52	542.84	533.60
Crystal system, space group	*P*2_1_/*c*	*C*2/*c*	*C*2/*c*	*C*2/*c*
*a* (Å)/*α* (°)	10.050(2)/90	19.917(4)/90	19.773(4)/90	20.003(4)/90
*b* (Å)/*β* (°)	7.4652(15)/105.83(3)	7.5116(15)/102.76(3)	7.4649(15)/102.51(3)	7.4692(15)/102.72(3)
*c* (Å)/*γ* (°)	7.4871(15)/90	7.5942(15)/90	7.5566(15)/90	7.6879(15)/90
Volume (Å^3^)	540.43(19)	1108.1(4)	1088.9(4)	1120.4(4)
*Z*, calculated density (g cm^–3^)	2, 2.955	4, 3.128	4, 3.311	4, 3.163
Absorption coefficient (mm^–1^)	4.945	4.973	5.439	4.729
*F*(000)	470	1020	1052	1040
Theta range for data collection	3.45 to 27.98°	2.91 to 28.06 °	2.93 to 27.97 °	2.92 to 28.06 °
Reflections collected/unique	6188/1285	5077/1324	5040/1294	6519/1307
Data/restraints/parameters	1285/17/108	1324/16/98	1294/0/98	1275/0/113
Goodness-of-fit on *F* ^2^	1.045	0.944	1.143	1.026
*R*_1_/W*R*_2_ [*I*>2*σ*(*I*)]	0.0278/0.0715	0.0333/0.0796	0.0350/0.0966	0.0251/0.0601
*R*_1_/W*R*_2_ (all data)	0.0318/0.0731	0.0460/0.0826	0.0375/0.0980	0.0352/0.0615

**Table 2:** Selected bond lengths [Å] for (NH_4_)_2_Co_3_(HPO_4_)_2_F_4_ (**1**), (NH_4_)Co_3_(HPO_4_)_2_(H_2_PO_4_)F_2_ (**2**), KCo_3_(HPO_4_)_2_(H_2_PO_4_)F_2_ (**3**), and KFe_3_(HPO_4_)_2_(H_2_PO_4_)F_2_ (**4**)

**1** ( NH_4_)_2_Co_3_(HPO_4_)_2_F_4_		**2** (NH_4_)Co_3_(HPO_4_)_2_(H_2_PO_4_)F_2_		**3** KCo_3_(HPO_4_)_2_(H_2_PO_4_)F_2_		**4** KFe_3_(HPO_4_)_2_(H_2_PO_4_)F_2_	
Co(1)–F(1)	2.0528(14)	Co(1)–F(1)	2.187(2)	Co(1)–F(1)	2.0731(19)	Fe(1)–F(1)	2.0938(19)
Co(1)–F(1)#	2.1448(15)	Co(1)–F(1)#5	2.082(2)	Co(1)–F(1)#1	2.179(2)	Fe(1)–F(1)#1	2.2419(18)
Co(1)–F(2)	2.0014(17)	Co(1)–O(1)	2.112(3)	Co(1)–O(4)	2.011(3)	Fe(1)–O(1)	**2.173(2)**
Co(1)–O(1)	2.1140(18)	Co(1)–O(2)#4	2.029(3)	Co(1)–O(1)	2.126(3)	Fe(1)–O(2)	2.132(2)
Co(1)–O(2)	2.121(2)	Co(1)–O(3)	2.026(3)	Co(1)–O(2)	2.094(3)	Fe(1)–O(4)	2.035(2)
Co(1)–O(3)	2.0716(19)	Co(1)–O(5)#3	2.116(3)	Co(1)–O(5)	2.021(3)	Fe(1)–O(5)	**2.034(2)**
Co(2)–F(1)#	2.0726(15)	Co(2)–F(1)	2.077(2)	Co(2)–F(1)#1	2.0634(19)	Fe(2)–F(1)#1	**2.0927(16)**
Co(2)–F(1)#	2.0726(15)	Co(2)–F(1)#1	2.077(2)	Co(2)–F(1)#4	2.0634(19)	Fe(2)–F(1)#4	**2.0927(16)**
Co(2)–O(1)	2.0461(19)	Co(2)–O(1)	2.059(3)	Co(2)–O(2)	2.050(2)	Fe(2)–O(1)	**2.141(2)**
Co(2)–O(1)#	2.0461(19)	Co(2)–O(1)#1	2.059(3)	Co(2)–O(2)#3	2.050(2)	Fe(2)–O(1)#3	**2.141(2)**
Co(2)–O(2)	2.1152(19)	Co(2)–O(5)#2	2.125(3)	Co(2)–O(1)	2.108(3)	Fe(2)–O(2)	**2.091(2)**
Co(2)–O(2)#	2.1152(19)	Co(2)–O(5)#3	2.125(3)	Co(2)–O(1)#3	2.108(3)	Fe(2)–O(2)#3	2.091(2)
P(1)–O(1)#4	1.5203(19)	P(1)–O(2)	1.504(3)	P(1)–O(1)	1.517(3)	P(1)–O(1)	1.513(2)
P(1)–O(2)	1.5218(19)	P(1)–O(5)	1.513(3)	P(1)–O(2)#13	1.517(2)	P(1)–O(2)#5	**1.513(2)**
P(1)–O(3)#5	1.5234(19)	P(1)–O(1)	1.517(3)	P(1)–O(3)	1.567(2)	P(1)–O(3)	1.570(2)
P(1)–O(4)	1.585(2)	P(1)–O(6)	1.565(3)	P(1)–O(4)#12	1.503(3)	P(1)–O(4)#2	1.501(2)
		P(2)–O(3))#6	1.491(3)	P(2)–O(5)#5	1.490(3)	P(2)–O(5)	1.492(2)
		P(2)–O(3)	1.491(3)	P(2)–O(5)	1.490(3)	P(2)–O(5)#6	1.492(2)
		P(2)–O(4) #6	1.569(3)	P(2)–O(6)#5	1.572(3)	P(2)–O(6)#6	1.571(2)
		P(1)–O(4)	1.569(3)	P(2)–O(6)	1.572(3)	P(2)–O(6)	**1.571(2)**

Symmetry transformations used to generate equivalent atoms:				
1:	#1 *x*, –*y* + 1/2, *z* – 1/2	#2 –*x* + 2, –*y* + 1, –*z* + 1	#3 –*x* + 2, *y* + 1/2, –*z* + 3/2	#4 –*x* + 2, *y* – 1/2, –*z* + 3/2
	#5 –*x* + 2, –*y*, –*z* + 1	#6 *x*, –*y* + 1/2, *z* + 1/2		
2:	#1–*x* – 1/2, –*y* + 1/2, –*z* – 1	#2 *x*, –*y*, *z* – 1/2	#3–*x* – 1/2, *y* + 1/2, –*z* – 1/2	#4 *x*, –*y* + 1, *z* – 1/2
	#5 *x*, –*y* + 1, *z* + 1/2	#6 –*x*, *y*, –*z* – 1/2	#7 –1/2, *y* – 1/2, –*z* – 1/2	
3:	#1 *x*, –*y* + 2, *z* – 1/2	#2 –*x* + 1, –*y* + 2, –*z* + 1	#3 –*x* + 1/2, –*y* + 3/2, –*z* + 1	#4 –*x* + 1/2, *y* – 1/2, –*z* + 3/2
	#5 –*x* + 1, *y*, –*z* + 3/2	#6 *x*, *y*, *z*–1	#7 *x* + 1/2, –*y* + 3/2, *z* – 1/2	#8 –*x* + 1,–*y* + 1, –*z* + 1
	#9 *x*, –*y* + 1, *z* – 1/2	#10 –*x* + 1/2, *y* – 1/2, –*z* + 1/2	#11 *x* + 1/2, *y* – 1/2, *z*	#12 –*x* + 1/2, –*y* + 5/2, –*z* + 1
	#13 –*x* + 1/2, *y* + 1/2, –*z* + 3/2	#14 *x* – 1/2, *y* + 1/2, *z*	#15 *x*, *y*, *z* + 1	#16 *x*, –*y* + 2, *z* + 1/2
4:	#1*x*, –*y*, *z* – 1/2	#2 –*x* + 1/2, –*y* + 1/2, –*z* + 1	#3–*x* + 1/2, –*y* – 1/2, –*z* + 1	#4 –*x* + 1/2, *y* – 1/2, –*z* + 3/2
	#5 –*x* + 1/2, *y* + 1/2, –*z* + 3/2	#6 –*x* + 1, *y*, –*z* + 3/2	#7 *x*, –*y*, *z* + 1/2	#8 –*x*, –*y*, –*z* + 1
	#9 *x* + 1/2, *y* – 1/2, *z* + 1	#10 *x* – 1/2, –*y* + 1/2, *z* – 1/2	#11 *x* – 1/2, *y* + 1/2, *z* – 1	#12 *x* – 1/2, –*y* – 1/2, *z* – 1/2
	#13 –*x*, *y*, –*z* + 1/2			

## Conclusions

Four metal fluorophosphates were synthesized with the ionothermal approach using the ionic liquids [C_4_py][PF_6_] (compounds **1** and **2**) and [C_4_mpy][PF_6_] (compounds **3** and **4**). Single crystal X-ray diffraction allowed unraveling of the unprecedented structure of these materials. Compound **1** is a macroanionic structure featuring {Co_3_(HPO_4_)_2_F_4_}^2–^ layers, while compounds **2–4** are isostructural framework structures featuring {M_3_(HPO_4_)_2_(H_2_PO_4_)F_2_}^–^ macroanions. Both structure types are built from face-sharing trimeric octahedron units interlinked to give corrugated layers with 3- and 6-membered rings. The resulting ring structure presents a Kagomé topology. All Co(2) cations are situated on special positions and form planar 6^3^ nets. The stacking sequence is AA for (compound **1**). and AA_*i*_ for (compounds **2–4**). In contrast to compound **1** PO_2_(OH)_2_ tetrahedra link the layers in the compounds **2–4** to form a 3D network. The second difference between the compound **1** and compounds **2–4** is that each Co(1) in compound **1** is coordinated by a terminal F anion, whereas compounds **2–4** is coordinated by O atom to link PO_2_(OH)_2_ tetrahedra. The third difference is that compound **1** comprises 2D layers stacked along [100], with hydrogen bond interactions between the ammonium groups and the terminal F and OH on the both sides of the layer, whereas compounds **2–4** constitute the channels within the *ab* plane of 10-rings along [001] formed by octahedron-trimer units interconnected by PO_2_(OH)_2_ tetrahedra along [100]. Comparing both structures, the neighboring corrugated layers with Kagomé topology of compounds **2–4** are shifted by |*b*|/2 parallel to [010]. In all compounds the negatively charged framework is compensated by K^+^ or NH_4_^+^ cations located between the structural layers.

TG experiments show that compounds **2–4** exhibit a one-step weight loss between 450 °C and 550 °C with the release of either H_2_O, NH_3_ and HF or H_2_O and HF. In contrast, compound **1** exhibits a two-step weight loss. In the first step between 300 °C and 400 °C only NH_3_ is released, whereas during the second between 530 °C and 570 °C, both water and HF are lost. Magnetic measurements revealed that all four compounds show long range magnetic order in their ground state and can be described as canted anti-ferromagnets.
