# Supplementary material for: Ionothermal synthesis of open-framework metal phosphates with a Kagomé lattice network exhibiting canted anti-ferromagnetism
Source: J Mater Chem C Mater. 2014 Aug 4;2(35):7417–27. doi: 10.1039/c4tc00290c (PMC4285085; doi:10.1039/c4tc00290c)
Supplement: Supplementary file 1 [file TC-002-C4TC00290C-s001.pdf]

Electronic Supplementary Material (ESI) for Journal of Materials Chemistry C.
This journal is © The Royal Society of Chemistry 2014

Supporting Information

Ionothermal Synthesis of Open-Framework Metal phosphates with a Kagomé Lattice Network exhibiting Canted Anti-Ferromagnetism

*Guangmei Wang[a], Martin Valldor[c], Bert Mallick[a], Anja-Verena Mudring[a,b]**

[a]Inorganic Chemistry III – Materials Engineering and Characterization, Ruhr-Universität Bochum, D-44780 Bochum

[b] Materials Science and Engineering, Iowa State University, Ames, IA 50011 USA and Critical Materials Institute, Ames Laboratory, Ames, IA 50011 USA

c Physics of Correlated Matter, Max-Plank-Institute for Chemical Physics of Solids, D-01187 Dresden.

Table 1a. Atomic coordinates (× 10^4) and equivalent isotropic displacement parameters (Å^2 ×10^3) for $(NH_4)_2Co_3(HPO_4)_2F_4$ (**1**). U(eq) is defined as one third of the trace of the orthogonalized Uij tensor.

	x	y	z	U(eq)
Co(1)	8181(1)	2276(1)	5724(1)	10(1)
Co(2)	10000	5000	5000	10(1)
P(1)	11569(1)	1168(1)	6850(1)	9(1)
O(1)	8780(2)	4867(2)	6782(3)	14(1)
O(2)	10323(2)	2308(3)	5894(3)	14(1)
O(3)	7782(2)	-233(2)	4515(2)	13(1)
O(4)	12756(2)	2456(3)	7978(3)	15(1)
F(1)	8275(1)	1253(2)	8300(2)	12(1)
F(2)	6157(2)	2647(2)	5396(2)	20(1)
N(1)	5298(3)	188(4)	7486(4)	23(1)

Table 1b. Atomic coordinates (× 10^4) and equivalent isotropic displacement parameters ($Å^2 \times 10^3$) for $(NH_4)Co_3(HPO_4)_2(H_2PO_4)F_2$ (**2**). U(eq) is defined as one third of the trace of the orthogonalized Uij tensor.

	x	y	z	U(eq)
Fe(1)	-7500	7432(1)	7675(1)	11(1)
Fe(2)	10000	10000	12565(1)	8(1)
Cl(1)	10000	10000	7927(1)	18(1)
Cl(2)	5000	5000	7357(1)	19(1)
O(1)	7500	6825(6)	9496(4)	19(1)
O(2)	9240(5)	8307(5)	11250(4)	30(1)
O(3)	7500	7963(7)	5862(4)	27(1)
O(5)	7500	5521(5)	4344(5)	31(1)
O(6)	9235(5)	8265(5)	3875(4)	35(1)
N(1)	7500	2634(7)	9681(6)	23(2)
N(2)	2500	7887(8)	5654(7)	26(1)
F(1)	7500	11077(3)	12584(4)	14(1)
P(1)	7500	7356(2)	10837(1)	9(1)
P(2)	7500	7613(2)	4501(2)	10(1)
O(4)	7500	5505(6)	1537(4)	37(1)

Table 1c. Atomic coordinates (× 10^4) and equivalent isotropic displacement parameters (Å^2 ×10^3) for $KCo_3(HPO_4)_2(H_2PO_4)F_2$ (**3**). U(eq) is defined as one third of the trace of the orthogonalized Uij tensor.

	x	y	z	U(eq)
Co(1	3401(1)	10208(1)	6932(1)	8(1)
Co(2	2500	7500	5000	7(1)
K(1)	5000	6518(2)	2500	27(1)
P(1)	1728(1)	11410(1)	5792(1)	6(1)
P(2)	5000	8732(2)	7500	9(1)
O(1)	2327(1)	10168(3)	5701(4)	14(1)
O(2)	3105(1)	7635(3)	7569(3)	13(1)
O(3)	1123(1)	10170(3)	6092(4)	12(1)
O(4)	3575(1)	12626(3)	5946(3)	14(1)
O(5)	4422(1)	9794(4)	7947(4)	20(1)
O(6)	5297(2)	7371(4)	9064(4)	21(1)
F(1)	3368(1)	11289(3)	9439(2)	11(1)

Table 1d. Atomic coordinates (× 10^4) and equivalent isotropic displacement parameters ($Å^2$ ×10^3) for $KFe_3(HPO_4)_2(H_2PO_4)F_2$ (**4**). U(eq) is defined as one third of the trace of the orthogonalized Uij tensor.

	x	y	z	U(eq)
Fe(1)	3416(1)	245(1)	6957(1)	10(1)
Fe(2)	2500	-2500	5000	10(1)
P(1)	1730(1)	1402(1)	5815(1)	9(1)
P(2)	5000	-1298(2)	7500	13(1)
F(1)	3380(1)	1320(2)	9450(2)	15(1)
O(1)	2331(1)	210(3)	5702(3)	16(1)
O(2)	3100(1)	-2356(3)	7593(3)	16(1)
O(3)	1144(1)	138(3)	6147(3)	14(1)
O(4)	3583(1)	2655(3)	5894(3)	17(1)
O(5)	4431(1)	-218(3)	7938(3)	22(1)
O(6)	5296(1)	-2649(3)	9043(3)	25(1)
K(1)	0	1507(2)	2500	31(1)

Table 2a. Hydrogen coordinates (x 10^4) and isotropic displacement parameters ($Å^2$ ×10^3) for for ($NH_4)_2Co_3(HPO_4)_2F_4$ (**1**)

	x	y	z	U(eq)
H(5)	12530(40)	3150(50)	8780(50)	48(13)
H(4)	4448(19)	-240(50)	7090(60)	64(16)
H(3)	5430(40)	930(40)	6620(30)	41(12)
H(2)	5470(30)	800(30)	8550(20)	18(8)
H(1)	5880(30)	-730(30)	7680(40)	25(9)

Table 2b. Hydrogen coordinates (x 10^4) and isotropic displacement parameters ($Å^2$ ×10^3) for for

$(NH_4)Co_3(HPO_4)_2(H_2PO_4)F_2$ (**2**)

	x	y	z	U(eq)
H(4)	734	2675	-368	33
H(6)	-1282	-775	-1872	20
H(1N)	-220	2050	-8591	25
H(2N)	322	656	-7799	25

Table 2c. Hydrogen coordinates (x 10^4) and isotropic displacement parameters ($Å^2$ ×10^3) for for $KCo_3(HPO_4)_2(H_2PO_4)F_2$ (**3**).

	x	y	z	U(eq)
H(3)	1245	9628	7052	19
H(6)	4978	6778	9300	32

Table 2d. Hydrogen coordinates (x 10^4) and isotropic displacement parameters ($Å^2$ ×10^3) for for $KFe_3(HPO_4)_2(H_2PO_4)F_2$ (**4**)

	x	y	z	U(eq)
H(6)	5763(13)	-2450(80)	9600(70)	9600(70)
H(3)	1260(40)	-710(80)	7010(80)	7010(80)

Table 3a.Hydrogen bonds [Å/°] for ($NH_4)_2Co_3(HPO_4)_2F_4$ (**1**)

D-H...A	d(D-H)	d(H...A)	d(D...A)	<(DHA)
O(4)-H(5)...O(3)#3	0.874(19)	1.84(2)	2.711(3)	174(5)
N(1)-H(4)...O(3)#7	0.883(13)	2.26(2)	3.049(3)	148(3)
N(1)-H(4)...F(2)#7	0.883(13)	2.54(4)	3.091(3)	121(4)
N(1)-H(3)...F(2)	0.891(13)	1.838(16)	2.702(3)	163(3)
N(1)-H(2)...F(2)#6	0.894(13)	1.791(16)	2.660(3)	163(3)
N(1)-H(1)...O(4)#4	0.885(13)	2.081(18)	2.911(3)	156(3)

Symmetry transformations used to generate equivalent atoms: #1 x,-y+1/2,z-1/2 #2 -x+2,-y+1,-z+1 #3 -x+2,y+1/2,-z+3/2 #4 -x+2,y-1/2,-z+3/2 #5 -x+2,-y,-z+1 #6 x,-y+1/2,z+1/2 #7 -x+1,-y,-z+1

Table 3b.Hydrogen bonds [Å/°] for $(NH_4)Co_3(HPO_4)_2(H_2PO_4)F_2$ (**2**)

D-H...A	d(D-H)	d(H...A)	d(D...A)	<(DHA)
O(4)-H(4)...F(1)#6	0.90	1.94	2.816(4)	165.7
O(6)-H(6)...O(2)#2	0.95	2.00	2.929(4)	163.3
O(6)-H(6)...F(1)#8	0.95	2.59	3.140(4)	117.3
N(1)-H(1N)...O(4)#9	0.97	2.28	2.912(3)	122.5
N(1)-H(1N)...O(2)#9	0.97	2.42	2.959(3)	115.1
N(1)-H(2N)...O(6)#10	0.93	2.09	2.977(3)	157.7

Symmetry transformations used to generate equivalent atoms: #1 -x-1/2,-y+1/2,-z-1 #2 x,-y,z-1/2 #3 -x-1/2,y+1/2,-z-1/2 #4 x,-y+1,z-1/2 #5 x,-y+1,z+1/2 #6 -x,y,-z-1/2 #7 -x-1/2,y-1/2,-z-1/2 #8 x,-y,z+1/2 #9 x,y,z-1 #10 -x,-y,-z-1

Table 3c.Hydrogen bonds [Å/°] for $KCo_3(HPO_4)_2(H_2PO_4)F_2$ (**3**)

D-H...A	d(D-H)	d(H...A)	d(D...A)	<(DHA)
O(3)-H(3)...O(4)#4	0.82	2.10	2.897(4)	163.7
O(6)-H(6)...O(3)#4	0.82	2.45	3.233(4)	160.6

Symmetry transformations used to generate equivalent atoms: #1 x,-y+2,z-1/2 #2 -x+1,-y+2,-z+1 #3 -x+1/2,-y+3/2,-z+1 #4 -x+1/2,y-1/2,-z+3/2 #5 -x+1,y,-z+3/2 #6 x,y,z-1 #7 x+1/2,-y+3/2,z-1/2 #8 -x+1,-y+1,-z+1 #9 x,-y+1,z-1/2 #10 -x+1/2,y-1/2,-z+1/2 #11 x+1/2,y-1/2,z #12 -x+1/2,-y+5/2,-z+1 #13 -x+1/2,y+1/2,-z+3/2 #14 x-1/2,y+1/2,z #15 x,y,z+1 #16 x,-y+2,z+1/2

Table 3d.Hydrogen bonds [Å/°] for $KFe_3(HPO_4)_2(H_2PO_4)F_2$ (**4**)

D-H...A	d(D-H)	d(H...A)	d(D...A)	<(DHA)
O(6)-H(6)...F(1)#14	0.95(2)	1.91(3)	2.827(3)	162(5)
O(3)-H(3)...O(4)#4 0	0.91(2)	1.99(2)	2.892(3)	170(7)

Symmetry transformations used to generate equivalent atoms: #1 x,-y,z-1/2 #2 -x+1/2,-y+1/2,-z+1 #3 -x+1/2,-y-1/2,-z+1 #4 -x+1/2,y-1/2,-z+3/2 #5 -x+1/2,y+1/2,-z+3/2 #6 -x+1,y,-z+3/2 #7 x,-y,z+1/2 #8 -x,-y,-z+1 #9 x+1/2,y-1/2,z+1 #10 x-1/2,-y+1/2,z-1/2 #11 x-1/2,y+1/2,z-1#12 x-1/2,-y-1/2,z-1/2 #13 -x,y,-z+1/2 #14 -x+1,-y,-z+2

Figure. S1 Experimental and simulated X-ray powder diffraction patterns $(NH_4)_2Co_3(HPO_4)_2F_4$ (1), $(NH_4)Co_3(HPO_4)_2(H_2PO_4)F_2$ (2), $KCo_3(HPO_4)_2(H_2PO_4)F_2$ (3), and $KFe_3(HPO_4)_2(H_2PO_4)F_2$ (4)

Figure S2 The IR spectra of $(NH_4)_2Co_3(HPO_4)_2F_4$ (1), $(NH_4)Co_3(HPO_4)_2(H_2PO_4)F_2$ (2), $KCo_3(HPO_4)_2(H_2PO_4)F_2$ (3), and $KFe_3(HPO_4)_2(H_2PO_4)F_2$ (4)

Figure S3 TG curves of $(NH_4)_2Co_3(HPO_4)_2F_4$ (1), $(NH_4)Co_3(HPO_4)_2(H_2PO_4)F_2$ (2), $KCo_3(HPO_4)_2(H_2PO_4)F_2$ (3), and $KFe_3(HPO_4)_2(H_2PO_4)F_2$ (4)

Figure S4 Thermal ellipsoid plot (50% probability) and atomic labeling scheme of $(NH_4)_2Co_3(HPO_4)_2F_4$ (1), $(NH_4)Co_3(HPO_4)_2(H_2PO_4)F_2$ (2), $KCo_3(HPO_4)_2(H_2PO_4)F_2$ (3), and $KFe_3(HPO_4)_2(H_2PO_4)F_2$ (4)

Figure S5 χT-T curves of $(NH_4)_2Co_3(HPO_4)_2F_4$ (1), $(NH_4)Co_3(HPO_4)_2(H_2PO_4)F_2$ (2), $KCo_3(HPO_4)_2(H_2PO_4)F_2$ (3), and $KFe_3(HPO_4)_2(H_2PO_4)F_2$ (4)

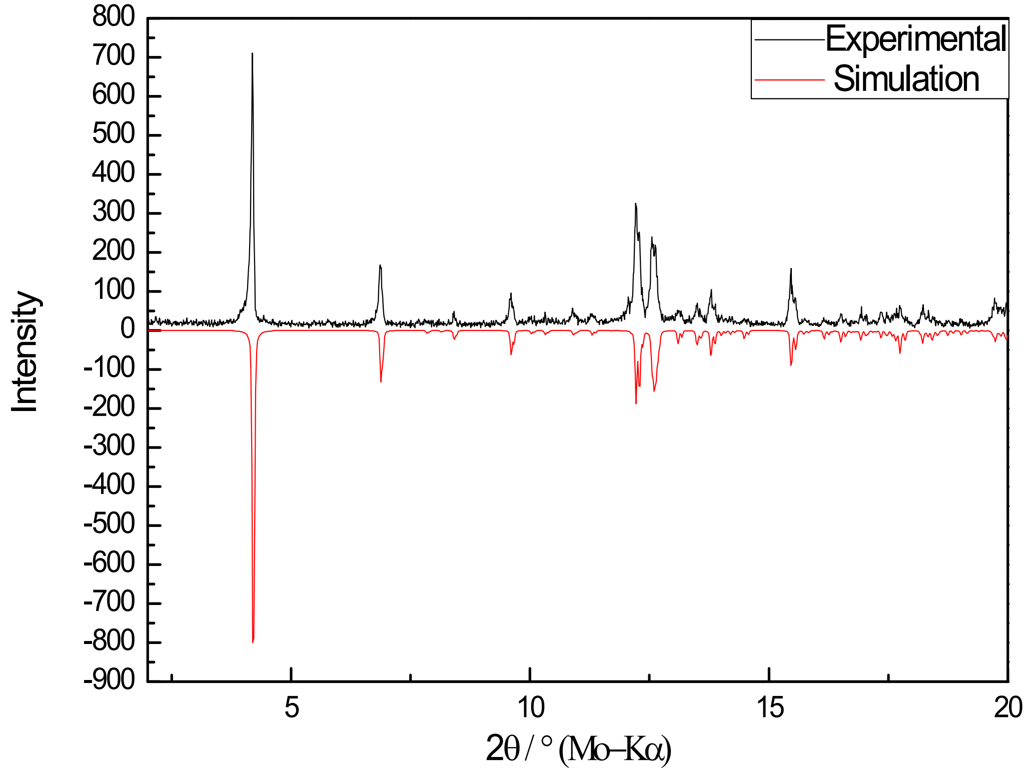

Figure S1a

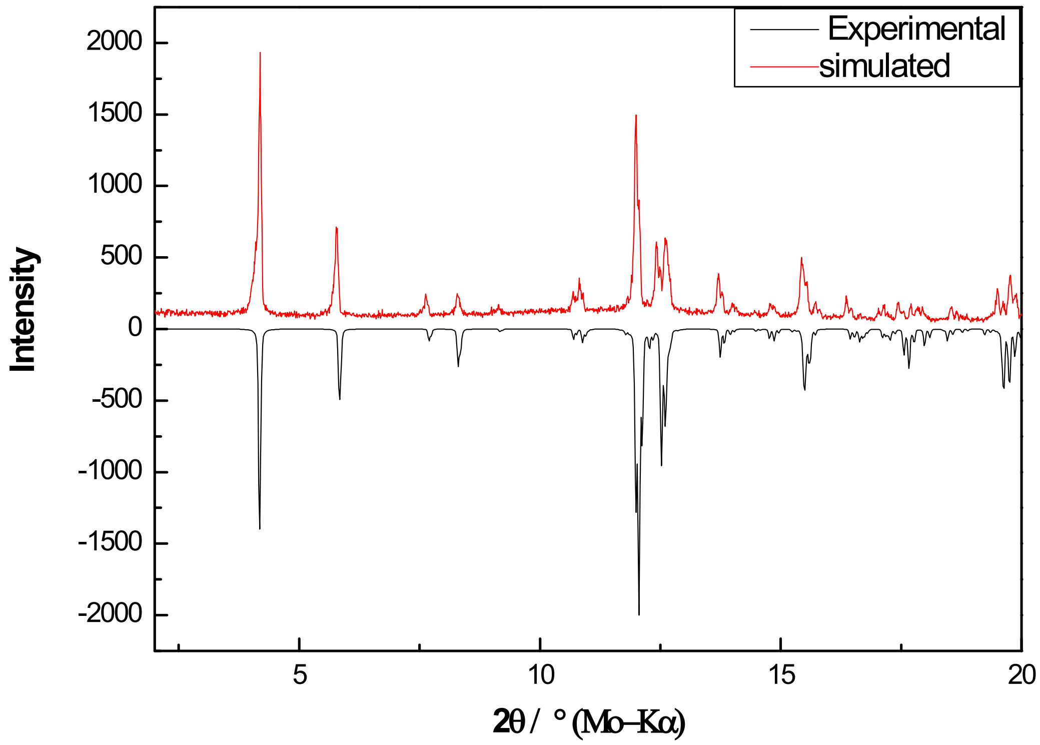

Figure S1b

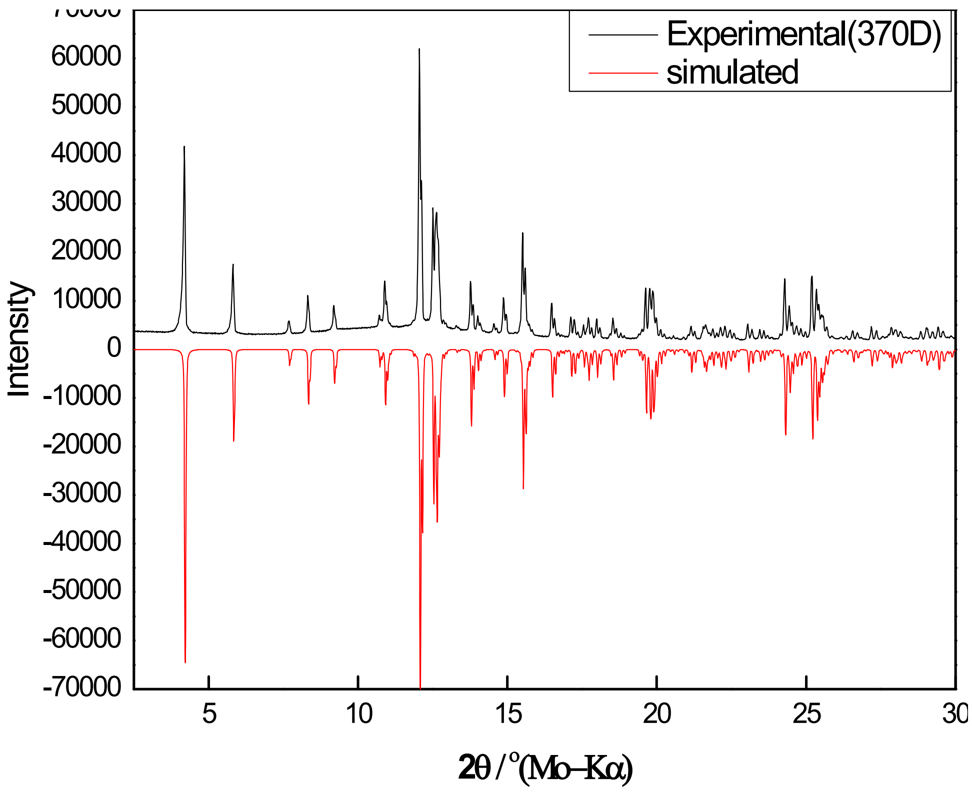

Figure S1c

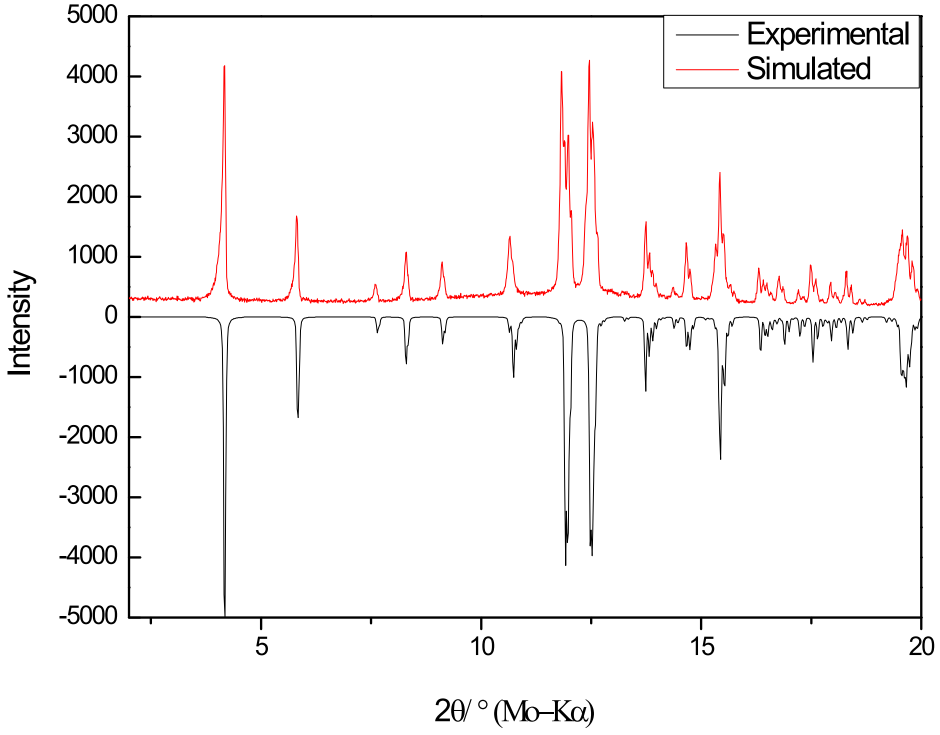

Figure S1d

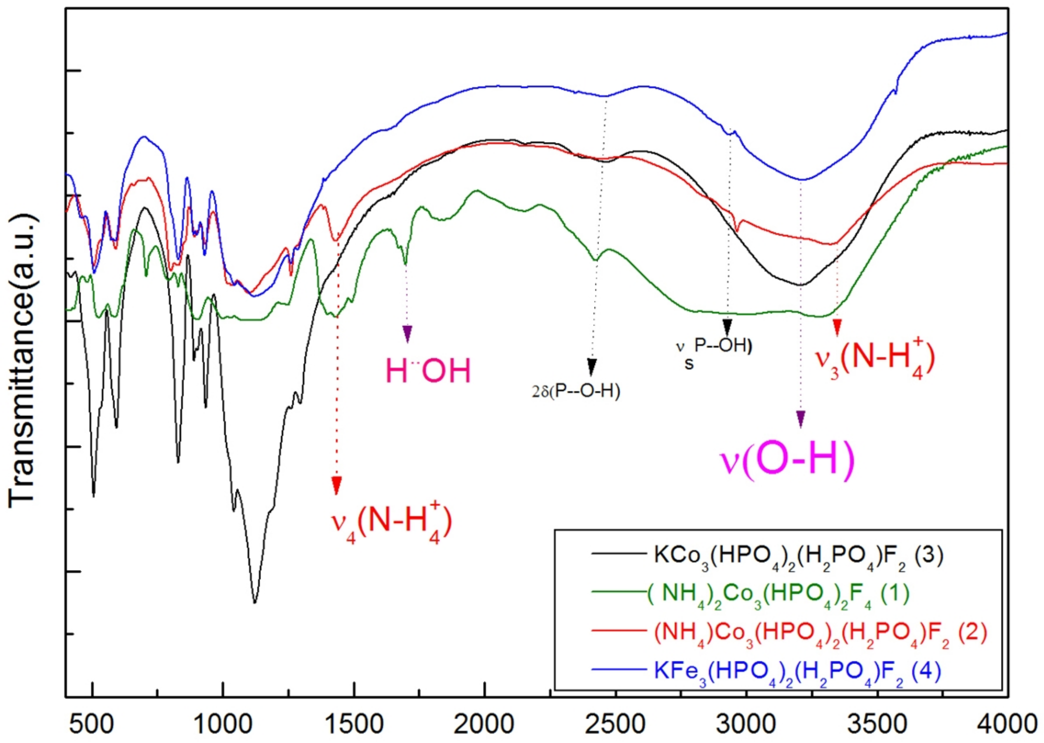
Transmittance(a.u.)
500
1000
1500
2000
2500
3000
3500
4000
H¨OH
2δ(P--O-H)
νs P--OH)
ν3(N-H4+)
ν(O-H)
ν4(N-H4+)
KCo3(HPO4)2(H2PO4)F2 (3)
(NH4)2Co3(HPO4)2F4 (1)
(NH4)Co3(HPO4)2(H2PO4)F2 (2)
KFe3(HPO4)2(H2PO4)F2 (4)

Figure S2

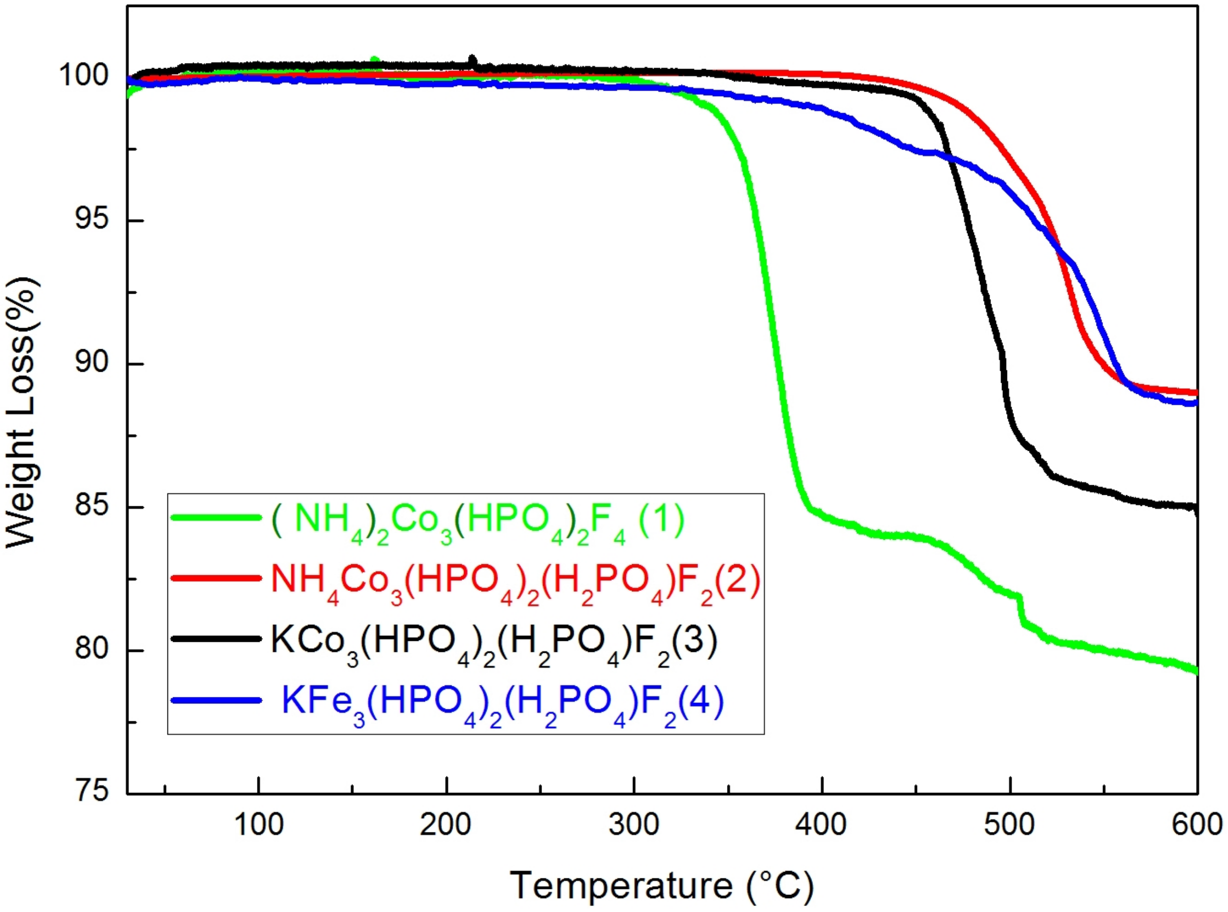
Weight Loss(%)
Temperature (°C)
100
95
90
85
80
75
100
200
300
400
500
600
(NH4)2Co3(HPO4)2F4 (1)
NH4Co3(HPO4)2(H2PO4)F2(2)
KCo3(HPO4)2(H2PO4)F2(3)
KFe3(HPO4)2(H2PO4)F2(4)

Figure S3

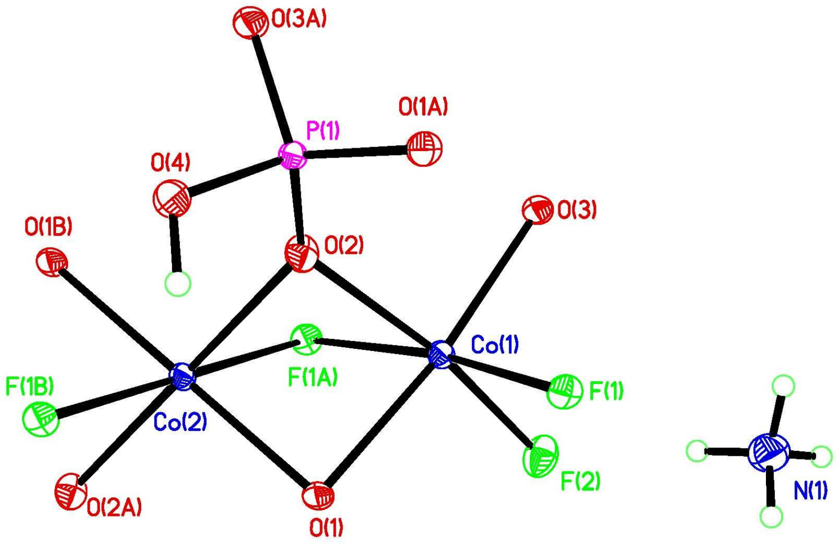

Figure S4a

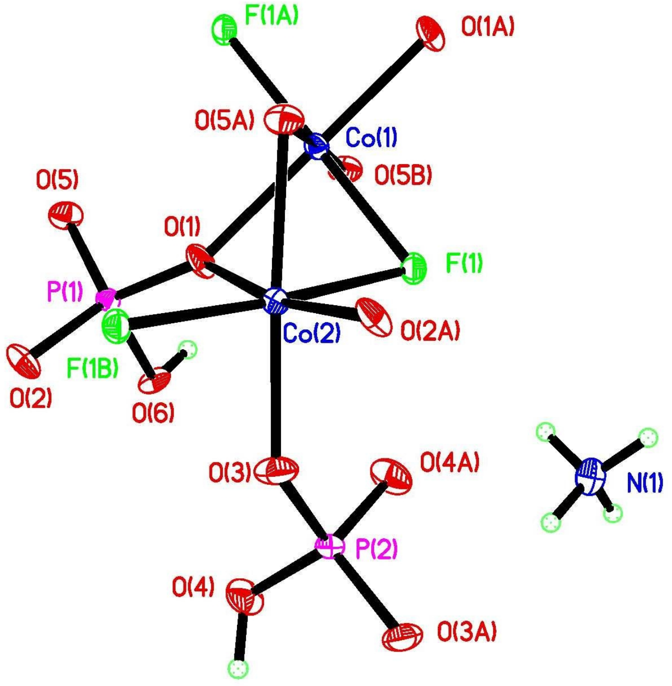

Figure S4b

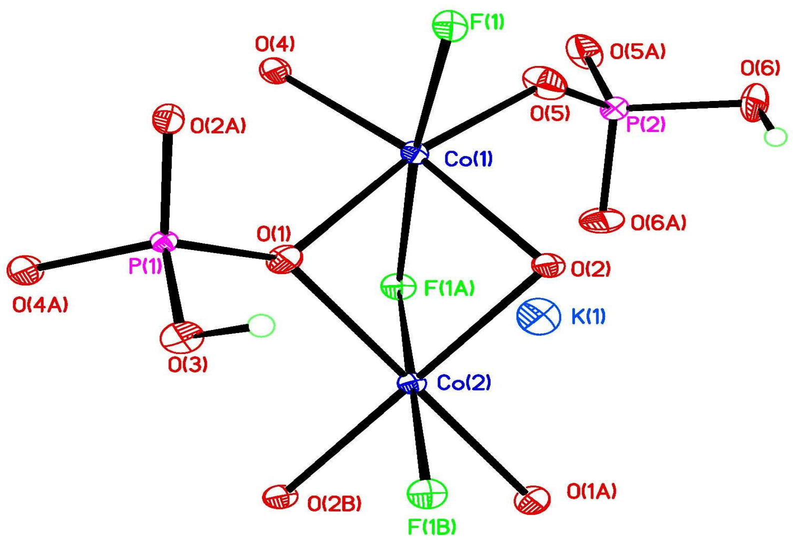

Figure S4c

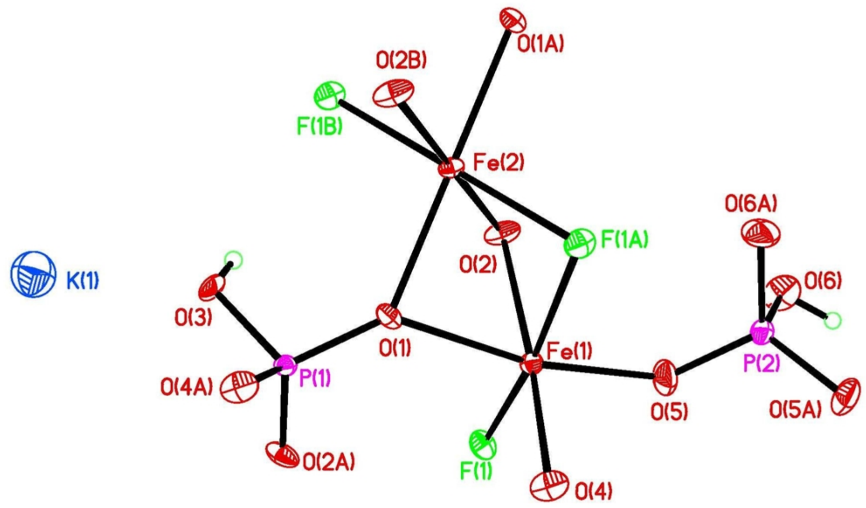

Figure S4d

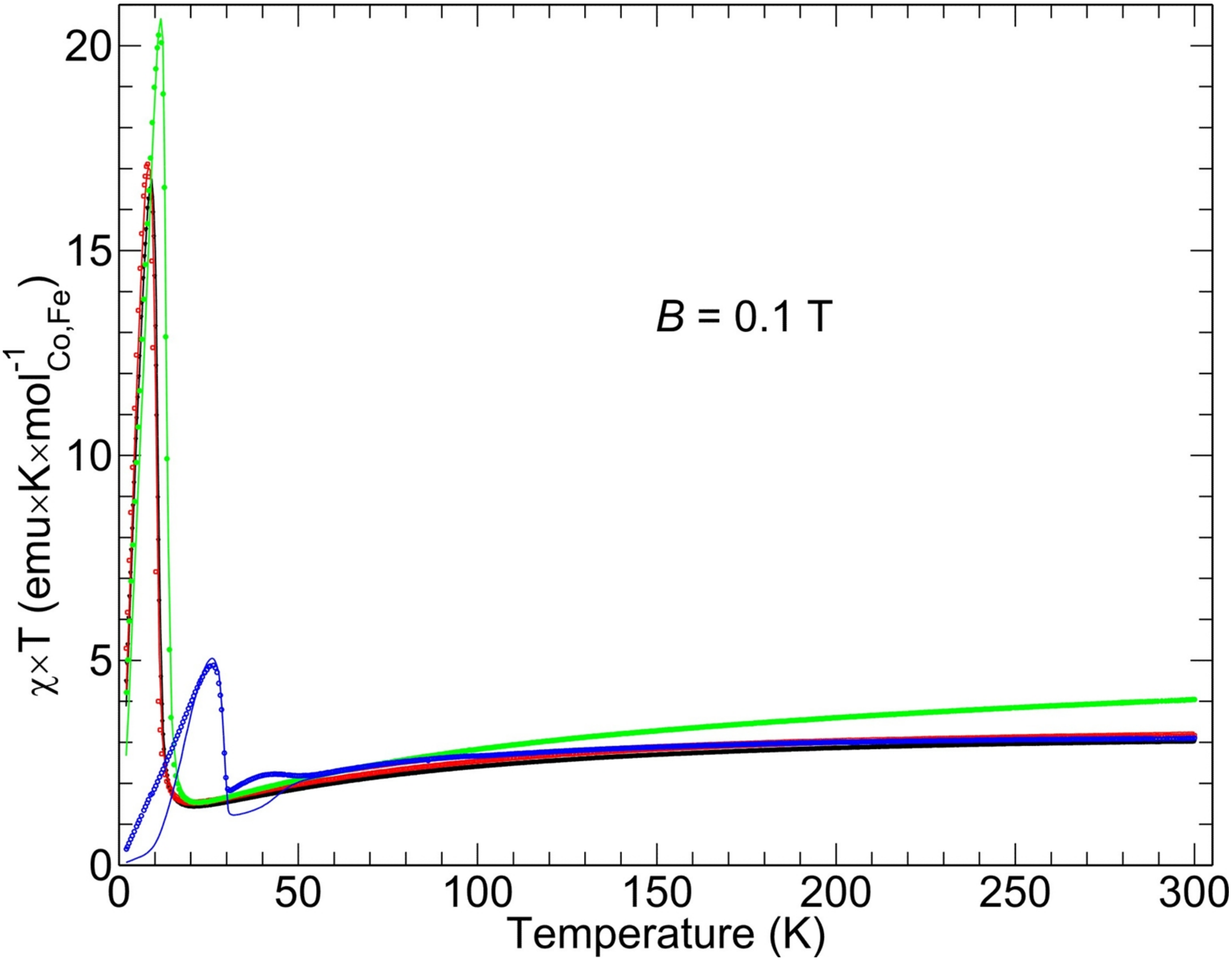

B = 0.1 T
χ×T (emu×K×mol$^{-1}_{Co,Fe}$)
Temperature (K)
0
5
10
15
20
0
50
100
150
200
250
300

Figure S5
